# Miglustat ameliorates isoproterenol-induced cardiac fibrosis via targeting UGCG

**DOI:** 10.1186/s10020-025-01093-w

**Published:** 2025-02-11

**Authors:** Jing Liu, Wenqi Li, Ran Jiao, Zhigang Liu, Tiantian Zhang, Dan Chai, Lingxin Meng, Zhongyi Yang, Yuming Liu, Hongliang Wu, Xiaoting Gu, Xiaohe Li, Cheng Yang

**Affiliations:** 1 State Key Laboratory of Medicinal Chemical Biology, College of Pharmacy, Nankai University, Haihe Education Park, 38 Tongyan Road, Tianjin, 300353 China; 2 Tianjin Key Laboratory of Molecular Drug Research, International Joint Academy of Biomedicine, Tianjin, 300457 China; 3 Department of Anesthesiology, National Cancer Center/National Clinical Research Center for Cancer/Cancer Hospital, Chinese Academy of Medical Sciences and Peking Union Medical College, No. 17 Nanli, Panjiayuan, Chaoyang District, Beijing, China

**Keywords:** β-Adrenergic receptor, Cardiac fibrosis, Miglustat, ERK, STAT3, Akt, GSK3β signaling, UGCG

## Abstract

**Background:**

Cardiac fibrosis is significant global health problem, which is associated with numerous cardiovascular diseases, and ultimately leads to the progression to heart failure. β-adrenergic receptor (β-AR) overactivation play a role in the development of cardiac fibrosis. Miglustat (Mig) has shown anti-fibrosis effects in multiple fibrotic diseases. However, it is unclear whether and how Mig can ameliorate cardiac fibrosis induced by β-AR overactivation.

**Methods:**

In vivo, mice were injected with isoproterenol (ISO) to induce cardiac fibrosis and treated with Mig. In vitro, primary cardiac fibroblasts were stimulated by ISO and treated with Mig. Levels of cardiac fibrosis, cardiac dysfunction, activation of cardiac fibroblasts were evaluated by real-time polymerase chain reaction, western blots, sirius red staining, immunohistochemistry staining and echocardiography. Through GEO data and knockdown UDP-glucose ceramide glycosyltransferase (UGCG) in primary cardiac fibroblasts, whether Mig alleviates cardiac fibrosis by targeting UGCG was explored.

**Results:**

The results indicated that Mig alleviated ISO-induced cardiac dysfunction. Consistently, Mig also suppressed ISO-induced cardiac fibrosis. Moreover, Mig attenuated ISO-induced cardiac fibroblasts (CFs) activation. To identify the protective mechanism of Mig on cardiac fibrosis, several classical β-AR downstream signaling pathways, including ERK, STAT3, Akt and GSK3β, were further analyzed. As expected, ISO activated the ERK, STAT3, Akt and GSK3β in both CFs and mouse hearts, but this effect was reversed pretreated with Mig. Besides, Mig ameliorates ISO-induced cardiac fibrosis by targeting UDP-glucose ceramide glycosyltransferase (UGCG) in CFs.

**Conclusions:**

Mig ameliorates β-AR overactivation-induced cardiac fibrosis by inhibiting ERK, STAT3, Akt and GSK3β signaling and UGCG may be a potential target for the treatment of cardiac fibrosis.

**Supplementary Information:**

The online version contains supplementary material available at 10.1186/s10020-025-01093-w.

## Introduction

Heart failure (HF) is a multifaceted clinical syndrome, and is a severe manifestation or late stage of various heart diseases (Mosterd et al. 2007). Cardiac fibrosis is a central pathological and physiological process, which modulates through extracellular matrix (ECM) stiffness and finally affects cardiac function of heart failure (Travers et al. 2016; Kuwahara et al. 2002). Moreover, myocardial fibrosis is also a kind of accompanied characteristics among advanced heart diseases, including myocardial infarction, dilated cardiomyopathy, as well as hypertension (Fan et al. 2023; Mandawat et al. 2021; Failer et al. 2022). Therefore, inhibiting cardiac fibrosis has become an important mean of slow down the progression of HF and other heart diseases.

Overactivation of the sympathetic nervous system plays a crucial role in the development of heart failure (Carter et al. 2015). After excessive activation of the sympathetic nervous system, the levels of catecholamines in the plasma increase, which participates the course of myocardial fibrosis due to multiple reasons (Cohn et al. 1990). ISO is a non-selective agonist of β-adrenergic receptor (β-AR) that can continuously activate β-AR and cause cardiac fibrosis. Previously, the model of ISO-induced cardiac fibrosis has been widely applied (Zhang et al. 2020; Xiao et al. 2018). β-blockers (including metoprolol) have beneficial effects on the treatment of cardiac fibrosis in patients with heart failure (Nuamnaichati et al. 2018). Based on this, this article attempts to find a drug that can significantly alleviate cardiac fibrosis, providing an effective theoretical basis for the treatment of cardiac fibrosis.

Miglustat (Mig) was launched in 2002 as a chemotherapy drug for the treatment of Gaucher's disease and Niemann Pick's disease type C. Mig is an inhibitor of targeting UDP-glucose ceramide glycosyltransferase (UGCG), which decreases the activity of UGCG, reduces the synthesis of glucose ceramides, and thereby inhibiting the synthesis of more complex Glycosphingolipids (GSLs) and preventing the accumulation of glycolytic enzyme volume (McCormack and Goa et al. 2003). Results has demonstrated that Mig alleviated bleomycin-induced pulmonary fibrosis mice (Nakamura et al. 2023). Meanwhile, other studies also showed that the therapeutic effect of Mig on CCl_4_-induced liver fibrosis (Iwanaga et al. 2023). These experimental findings prove that Mig has a good therapeutic effect on pulmonary and liver fibrosis, but its role in cardiac fibrosis is unknown.

UDP-glucose ceramide glycosyltransferase (UGCG) catalyzes the first glycosylation step in the biosynthesis of glycosphingolipids, which are membrane components containing lipid and sugar moieties. UGCG, which transfers UDP-glucose to ceramide to synthesize glucosylceramide (GlcCer) (Li et al. 2023), is located on the cytoplasmic side of the Golgi apparatus. Glucosylceramide (GlcCer) is the core structure of many glycosphingolipids (GSLs) (Ichikawa and Hirabayashi et al. 1998; Russo et al. 2018). Meanwhile, UGCG has various functions in heart. Firstly, lack of UGCG impaired the transport of β1-adrenergic receptors (Andersson et al. 2021). Besides, UGCG inhibition could prevent myocardial hypertrophy caused by chronic kidney disease (Baccam et al. 2022). Additionally, knock down UGCG ameliorates cardiac hypertrophy by mediating mitochondrial oxidative stress and ERK signaling pathway (Cui et al. 2023). Taken together, these results indicated that UGCG plays a significant role in heart. However, the role of UGCG in cardiac fibroblasts is still unclear.

Consequently, our study focused on whether Mig possessed protective effect on cardiac fibrosis and explored whether the mechanism of Mig is relevant to UGCG.

## Materials and methods

### Animals

Animals, which consist of male C57BL/6J mice (around 2 months old) and Sprague Dawley rats (born in 24h) were obtained from Weitonglihua (Beijing, China). Mice were fed in a SPF level condition in Nankai Animal Resources Center, which possessed free access to food and water. All animal experiments were conducted by the Institutional Animal Care and Use Committee (IACUC) of Nankai University (No. SYXK 2019‐0001).

The mice were randomly divided into 5 groups including CON group, ISO (10mg/kg; Sigma-Aldrich, I5627, USA) group, Mig (Aladdin, M137346, China) 150 mg/kg group (ISO + Mig 150 mg/kg), Mig 300 mg/kg group (ISO + Mig 300 mg/kg) and Met (MCE, HY-17503, China) 30 mg/kg group (ISO + Met 30 mg/kg). Among them, ISO group, Mig 150 mg/kg group, Mig 300 mg/kg group and Met 30 mg/kg group were subcutaneously injected with ISO (10 mg/kg) every day for 7 days (Wu et al. 2021), while CON group injected with equal saline. And Mig groups, including Mig 150 mg/kg group and Mig 300 mg/kg group, was orally twice a day for 7 days (Nakamura et al. 2023), and Met group (30 mg/kg) was orally as positive drug every day for 7 days (Li et al. 2023; Lai et al. 2021). After 24 h of the last ISO injection, mice were conducted echocardiographic measurements and sacrifice.

### Drug reagents

For drug reagents of animal studies, ISO (Sigma-Aldrich, I5627, USA), Mig (Aladdin, M137346, China) as well as Met (MCE, HY-17503, China) were all dissolved in saline. For drug reagents of cell studies, ISO (Sigma-Aldrich, I5627, USA) was dissolved in sterile water, while both Mig (Aladdin, M137346, China) and Met (MCE, HY-17503, China) were dissolved in DMSO. ISO (10 µM), Mig (100 µM and 200 µM) and Met (10 µM) were used in our research.

### Echocardiographic measurements

On days 8, the mice were examined by echocardiography after anesthetization through inhalation 1–2% isoflurane (Baxter Healthcare Corp., R510-22, USA). The Vevo 2100 system (VisualSonics Inc., Toronto, ON, Canada) with an MX400 (30MHz) probe was used to detect left ventricular inner dimension systolic (LVID; s), ventricular inner dimension diastolic (LVID;d), left ventricular posterior wall thickness in diastole (LVPW;d) left ventricular end diastolic volume (LVEDV), and left ventricular end systolic volume (LVESV), which were recorded using M and B mode images. Left ventricular ejection fraction (LVEF) (LVEF = (LVEDV-LVESV)/LVEDV*100%) was calculated at parasternal long-axis (PLAX) view.

### Histological analysis

After cardiac function was measured, the mice were sacrificed and their hearts removed after being washed with PBS. The cardiac tissues for histological analysis were placed in 4% paraformaldehyde for 24 h to fix, then embedded with paraffin and cut at a thickness of 5 μm. And we evaluated the extent of cardiac fibrosis through Sirius red staining (Sbjbio, BP-DL030, China), and the percentage of collagen area was measured using a quantitative digital image analysis system (Image-Pro Plus 6.0) to evaluate cardiac fibrosis.

### Isolation of cardiac fibroblasts and cardiomyocytes

Neonatal rat cardiac fibroblasts (NRCFs) and Neonatal rat cardiomyocytes (NRCMs) were isolated through enzymatic digestion from hearts of Sprague Dawley rats (born in 24h), based on the previous operation (Li et al. 2023). The mice were euthanized under the standard protocol, soaked in 75% alcohol for 5 min, and then the heart tissue was removed. The heart tissues were minced and digested with 0.025% (w/v) pancreatin (Gibco, 27250018, USA) and 0.08% collagenase type II (Gibco, 17101015, USA) in Hank’s Balanced Salt Solution (HBSS, without Ca2 + , Mg2 +) (Thermo scientific, 88284, USA) at 37 °C for 30 min. The collected cells were spun down and resuspended in culture medium: DMEM (Solarbio, 12100500, China) with 20% FBS (ExCellBio, FSP500, China) and penicillin–streptomycin (Gibco, 15070063, USA). After washing out digestion buffer with culture medium twice, cells were seeded on cell culture dishes and incubated for 2 h in the incubator (37 °C, 5% CO2). NRCFs and NRCMs were collected and cultured by differential adhesion separation. NRCFs adhered to the dish within 0–2 h, and NRCMs began to adhere to the dish after overnight culture and appear spontaneous pulsation.

The passage 2 of NRCFs were used in our research. Before ISO treatment for 24 h, we cultured NRCFs without serum for 24 h, pretreated by Mig, Met or identical DMSO ahead for 1 h. The passage 0 of NRCMs were used in our research. Before ISO treatment for 24 h, we cultured NRCMs without serum for 1 h, pretreated by Mig, Met or identical DMSO ahead for 1h.

### CCK-8 assay

We cultured NRCFs with 5000 cells per well. After drug incubation, CCK-8 (Dojindo, CK04, Japan) were equally added into 96-well plates. After cell incubation for 3–4 h, the OD450nm was revealed by Microplate Reader (Bio-Rad, Hercules, USA).

### Cell transfection

NRCFs were transfected using RNAi MAX transfection reagent (Thermo, 13778030, USA) for siRNA. After transfection for six hours, the cells were placed under normal culture conditions and grown for 24 h. To understand the role of UGCG in the pathological state of cardiac fibroblasts, UGCG knockdown was performed on the cells. Introduce siRNA (sense sequence 5′-GCGAAUCCAUGACAAUAUACATT-3′; nonsense strand 5′-UGUAUAUUGUCAUGGAUUCGCGTT-3′) into cells, place in serum-free culture medium for 24 h, and then act under ISO stimulation for 24 h.

### Total RNA extraction and real-time PCR

We extracted the total RNA from frozen rat cardiac tissue and cells with Trizol Reagent (Invitrogen, 10296028CN, USA) and FastKing gDNA SuperMix (TianGen, KR118, China). Quantitative Real-time PCR (QRT-PCR) was conducted through Hieff UNICON qPCR SYBR Mix (Yeasen, 11198ES08, China). The expression of Collagen I (Col-I), Collagen III (Col-III), Fibronectin (Fn), Connective Tissue Growth Factor (CTGF), Proliferating Cell Nuclear Antigen (PCNA), Alpha Smooth Muscle Actin (α-SMA), Atrial Natriuretic Peptide (ANP) and Brain Natriuretic Peptide (BNP) were then determined based on protocols. We applied GAPDH as normalization. The sequences of QRT-PCR are in Table 1.

**Table 1:** Primers for QRT-PCR

Gene	Forward primer sequence (5′-3′)	Reverse primer sequence (3′-5′)
Col-I	CCAAGAAGACATCCCTGAAGTCA	TGCACGTCATCGCACACA
Col-III	TGGTCCTCAGGGTGTAAAGG	GTCCAGCATCACCTTTTGGT
Fn	GTGTAGCACAACTTCCAATTACGAA	GGAATTTCCGCCTCGAGTCT
CTGF	CCAACTATGATTAGAGCCAACTG	AGGCACAGGTCTTGATGAAC
PCNA	CATCCGCGCTTTCTGATTGG	TCCGGAGCACTCATTTTCCC
α-SMA	GCTGGTGATGATGCTCCCA	GCCCATTCCAACCATTACTCC
ANP	CTTCCAGGCCATATTGGAG	GGGGGCATGACCTCATCTT
BNP	ACAAGATAGACCGGATCGGA	AGCCAGGAGGTCTTCCTACA
UGCG	AGTGATCTGGGGAAGGAGCA	GCCTTGCAATCCTGTCTGTC
GAPDH	AGGTCGGTGTGAACGGATTTG	GGGGTCGTTGATGGCAACA

### Western blot

Proteins were extracted from cardiac tissue and cell samples by RIPA Lysis Buffer (Beyotime Biotechnology, P0013C, China). BCA assay (Beyotime Biotechnology, China) was performed to normalize the proteins. 10% SDS–PAGE Gel Assay (Yazyme Bio-technology, PG113, China) was used to study different proteins. Then, proteins were transferred to a PVDF membrane. After blocking at room temperature for 1 h with 5% milk powder, the membrane was incubated with primary antibody at 4 ℃ overnight and incubated HRP-conjugated secondary antibody (Abacm, ab6789, USA) on the next day. We visualized proteins with chemiluminescence system (Syngene, Affinity, USA). We applied β-Tubulin and GAPDH as normalization. Image J was used to quantitative analysis. The primary antibodies are in Table 2.

**Table 2:** The antibodies for Western Blot

Antibody	Company	No
Col-I	Cell Signaling Technology	72026S
Col-III	Santa Cruz Biotechnology	sc-271249
Fn	Proteintech	15613-AP
CTGF	Santa Cruz Biotechnology	sc-365970
PCNA	Santa Cruz Biotechnology	sc-56
α-SMA	Affinity	AF1032
p-ERK1/2	Affinity	AF1015
ERK1/2	Affinity	AF0155
p-STAT3	Affinity	AF3293
STAT3	Affinity	AF6294
p-Akt	Affinity	AF0016
Akt	Affinity	AF6261
p-GSK3β	Affinity	AF2016
GSK3β	Affinity	AF5016
UGCG	Santa Cruz Biotechnology	sc-293235
β-Tubulin	Affinity	AF7011
GAPDH	Affinity	AF7021
Bcl-2	Affinity	AF6139
Bax	ABclonal	A7626
Smad2	Affinity	AF6449
p-Smad2	Affinity	AF3449
Smad3	Affinity	AF6362
p-Smad3	Affinity	AF3362

### Immunofluorescence

For tissue samples, sections were deparaffinized, the endogenous peroxidase of the heart tissue was inactivated, and antigen was retrieved before analysis. Sections were stained with antibodies against UGCG (1:200; Abmart), p-ERK (1: 200; Affinity), cTnT (1:200; Santa Cruz) and α-SMA (1:200; Santa Cruz) at 4 ℃ overnight. The second day, the sections were incubated with TRITC or FITC conjugated secondary antibodies FITC-conjugated (Solarbio, SF134, China) or TRITC‑conjugated (Jackson Immuno Research, 111–025-003, USA) secondary antibodies at for 1 h at 37 ℃ in the dark.

For cell samples, cells were fixed with 4% paraformaldehyde (Solarbio, P1110, China) for 10 min, washed with PBS, permeabilized with 0.2% Triton X100 (Solarbio, T8200, China) in PBS for 10 min, blocked with 5% bovine serum albumin (BSA, Solarbio, A8020, China), and incubated with anti‑UGCG (1:200; Santa Cruz), anti‑p-ERK (1: 200; Affinity), anti‑α-SMA (1:200; Santa Cruz) and anti‑p-GSK3β (1:200; Affinity) antibodies overnight at 4 °C. Cells were washed and incubated with FITC-conjugated (Solarbio, SF134, China) or TRITC‑conjugated (Jackson Immuno Research, 111–025-003, USA) secondary antibodies for 2 h at room temperature in the dark. After washing with PBS three times, the slices were mounted with anti-fluorescence attenuation tablets (including 4’,6‑diamidino‑2‑phenylindole, DAPI) (Solarbio, S2110, China). Immunofluorescence was observed under a microscope digital camera system (Zeiss, LSM 800 with Airyscan, Germany). ZEN 3.5 was used to import image. Image J was used to quantitative analysis.

### Immunohistochemistry

For immunohistochemical staining, sections were deparaffinized and antigen retrieved. Sections were stained with antibodies against α-SMA (Santa Cruz Biotechnology) and Col-I (Cell Signaling Technology) at 4 ℃ overnight, and the following steps were performed according to the instructions of the immunohistochemirtry (IHC) detection kit (ABclonal, RK05872, China).

### GEO datasets analysis

We first downloaded the expression matrix, sample grouping, and platform information of the GSE135055 dataset from GEO (https://www.ncbi.nlm.nih.gov/geo/). Then, we used the limma R package for data quality control and differential analysis. The threshold was set to |log2Fold Change|> 1 and adjusted P < 0.05 for differentially expressed genes. Visualization was performed using the ggplot2 R package. The expression of UGCG in the left ventricular induced by heart failure were analyzed in GSE135055, including HF (n = 9) as well as healthy control (n = 21). Then, the correlation between UGCG and cardiac fibrosis progression was validated.

### Statistical analysis

All experimental data, which were shown as Mean ± SEM, were performed and analyzed through Prism 9.0. All data were conducted by normality tests. The difference among two groups was conducted by two-tailed Student’s t-test. The difference between more than two groups was conducted by one-way analysis of variance ANOVA with Tukey’s post-hoc multiple comparison test. P < 0.05 was considered statistical significance.

## Results

### Mig alleviates ISO-induced cardiac dysfunction in vivo

**Fig. 1:**
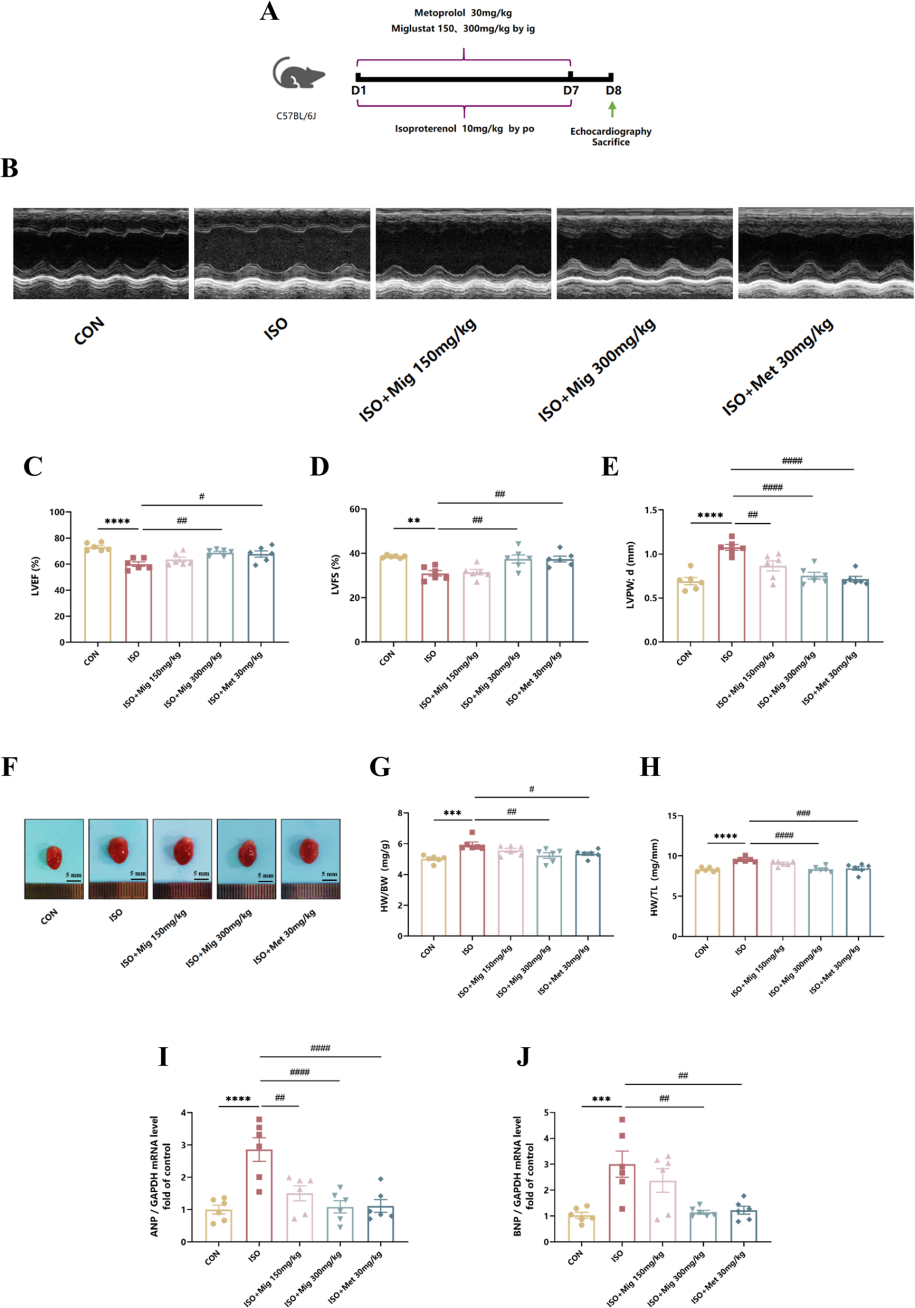
The effect of Mig on ISO-induced cardiac dysfunction. **A** Schematic representation of the animal model. **B** Representative echocardiographic M mode images on day 8. **C** Echocardiographic level of LVEF (%) (n = 6 per group). **D** Echocardiographic level of LVFS (%) (n = 6 per group). **E** Echocardiographic level of LVPW;d (n = 6 per group). **F** Representative images of heart size. Scale bar: 5 mm. **G** Quantitative analysis of HW/BW ratio (n = 6 per group). **H** Quantitative analysis of HW/TL ratio (n = 6 per group). **I** The expression of ANP in heart tissues by QRT-PCR (n = 6 per group). **J** The expression of in heart tissues by QRT-PCR (n = 6 per group). Met was used as positive control. All the genes were normalized to GAPDH. The data were presented by Mean ± SEM, and analyzed using one-way ANOVA with Tukey’s post-hoc multiple comparison test. **, P < 0.01, ***, P < 0.001, ****, P < 0.0001 vs. CON; #, P < 0.05, ##, P < 0.01, ###, P < 0.001, ####, P < 0.0001 vs. ISO. *LVPW*, d left ventricular posterior wall thickness, *LVEF* left ventricular ejection fraction, *LVFS* left ventricular fractional shortening, *HW/BW* the ratio of heart weight to body weight, *HW/TL* the ratio of heart weight to tibia length

In this study, we demonstrated that sustained stimulation of β-ARs significantly induced cardiac dysfunction and synthesis and secretion of growth factors. Cardiac dysfunction is one of the main pathological manifestations of cardiac fibrosis (Travers et al. 2016; Kuwahara et al. 2002). To investigate the effects of the Mig on cardiac function, echocardiography was performed in all groups. Echocardiography showed poor cardiac performance in the ISO group (Fig. 1B). Firstly, we investigated the LVPW;d, LVESV and LVEDV, which are important parameters reflecting cardiac function. LVESV and LVEDV were both significantly reduced in ISO mice, whereas Mig also partly restored LVESV and LVEDV (Figure S2). LVEF and LVFS were lower in ISO mice than that in CON mice, while Mig treatment improved the attenuated LVEF and LVFS (Fig. 1C, D).

To determine whether ISO induced cardiac hypertrophy, the left ventricular posterior wall thickness in diastole (LVPWd) was measured. LVPW;d was significantly increased in ISO mice, whereas Mig partly restored LVPW;d (Fig. 1E). Morever, the heart size of ISO mice was larger than that in CON mice, while Mig attenuated this adverse change (Fig. 1F). Furthermore, the ratio of heart weight to body weight (HW/BW) and the ratio of heart weight to tibia length (HW/TL), which are the key indicator of cardiac fibrosis, were up-regulated in ISO treated-mice, while Mig reversed this process (Fig. 1G, H). Besides, ANP and BNP, which were vital indicators of heart failure, demonstrated the same results (Fig. 1I, J). In summary, these data demonstrated that Mig alleviated ISO-induced cardiac dysfunction.

### Mig suppresses ISO-induced cardiac fibrosis in vivo

**Fig. 2:**
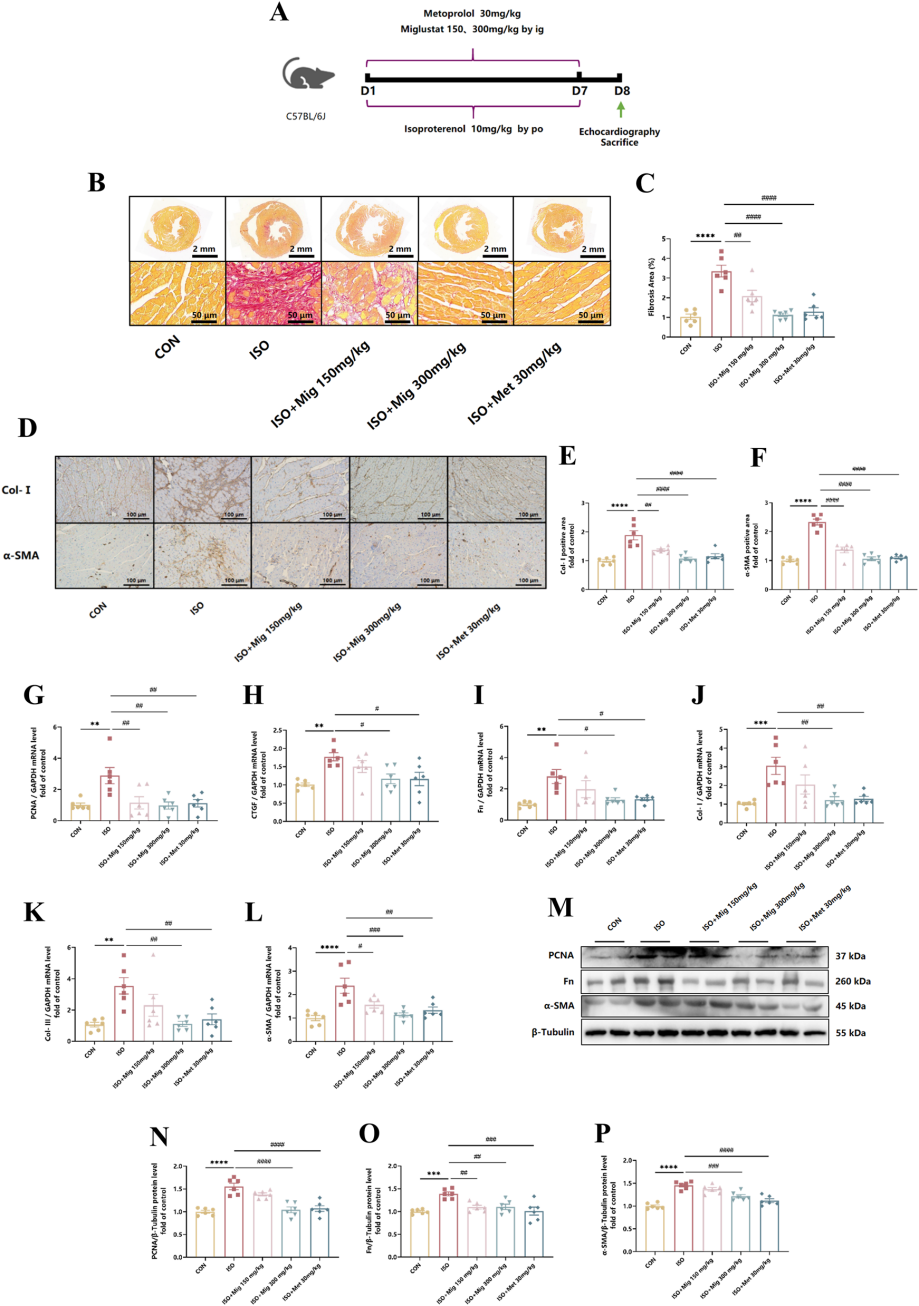
The effect of Mig on ISO-induced cardiac fibrosis. **A** Schematic representation of the animal model. **B** Representative 1 × and 20 × images of picrosirius red-stained in heart tissues. Scale bar (upper): 2 mm, scale bar (lower): 50 μm. **C** Quantification of picrosirius red-stained in heart tissues (n = 6 per group). **D** Representative 20 × images of Col-I and α-SMA immumohistochemical staining of heart tissues. Scale bar: 100 µm. **E** Quantification of Col-I immumohistochemical staining of heart tissues (n = 6 per group). **F** Quantification of α-SMA immumohistochemical staining of heart tissues (n = 6 per group). **G** The expression of PCNA in heart tissues by QRT-PCR (n = 6 per group). **H** The expression of CTGF in heart tissues by QRT-PCR (n = 6 per group). **I** The expression of Fn in heart tissues by QRT-PCR (n = 6 per group). **J** The expression of Col-I in heart tissues by QRT-PCR (n = 6 per group). **K** The expression of Col-III in heart tissues by QRT-PCR (n = 6 per group). **L** The expression of α-SMA in heart tissues by QRT-PCR (n = 6 per group). **M** Representative images of PCNA, Fn and α-SMA in heart tissues by Western Blot. **N** The expression of PCNA in heart tissues by Western Blot (n = 6 per group). **O** The expression of Fn in heart tissues by Western Blot (n = 6 per group). **P** The expression of α-SMA in heart tissues by Western Blot (n = 6 per group). Met was used as positive control. All the genes were normalized to GAPDH. The data were presented by Mean ± SEM, and analyzed using one-way ANOVA with Tukey’s post-hoc multiple comparison test. **, P < 0.01, ***, P < 0.001, ****, P < 0.0001 vs. CON; #, P < 0.05, ##, P < 0.01, ###, P < 0.001, ####, P < 0.0001 vs. ISO

To verify whether Mig played a role in cardiac fibrosis, Cardiac fibrosis in mice was quantitatively determined with different methods. The distribution of collagen fibres in heart tissues was visualized by staining with Picrosirius Red. Interestingly, fibrosis areas of heart tissues were markedly increased in ISO group, while Mig dose-dependently alleviated this process (Fig. 2B, C). Cardiac fibrosis resulted from the increased synthesis and decreased degradation of extracellular matrix (ECM), which is mainly composed of type I collagen (85%) (Heiden et al. 2010; Chen et al. 2012). Meanwhile, the positive areas of Col-I and α-SMA, which were conducted by immumohistochemical staining, were significantly reduced with Mig treatment, compared with that after isoprenaline administration (Fig. 2D–F). In addition, cardiac fibrosis markers, including PCNA, CTGF, Fn, Col-I, Col-III and α-SMA, also showed the similar results in both mRNA (Fig. 2G–L) and protein levels (Fig. 2K–P) in heart tissues. Taken together, these data proved that Mig suppressed ISO-induced cardiac fibrosis.

### Mig attenuates ISO-induced CFs activation in vitro

**Fig. 3:**
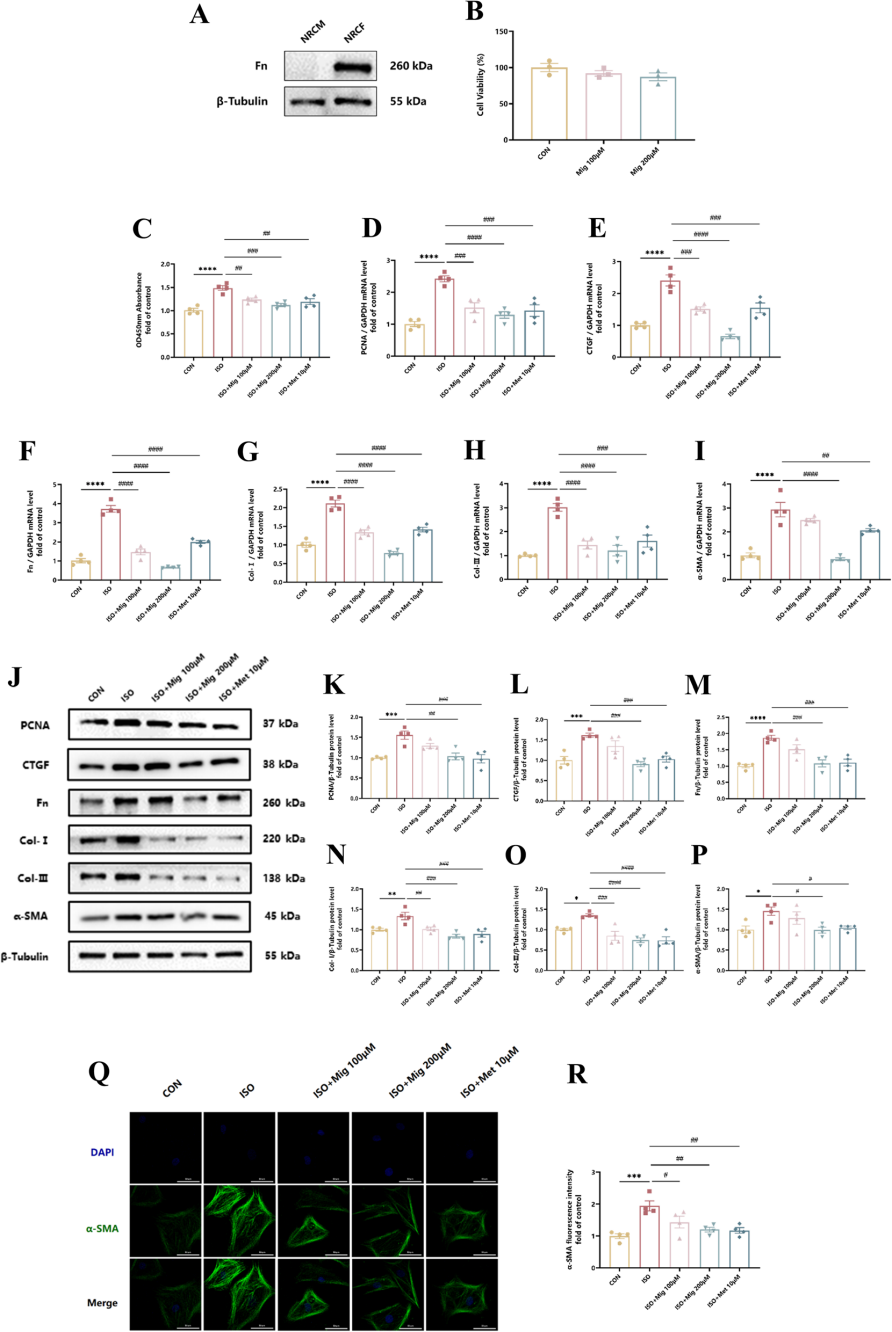
The effect of Mig on ISO-induced CFs activation. **A** The expression of Fn in NRCFs by Western Blot. **B** Cell toxicity of Mig in NRCFs (n = 3 per group). Mig (100 μM and 200 μM) were treated in NRCFs for 24h. **C** Cell viability of Mig in NRCFs (n = 4 per group). **D** The expression of PCNA in NRCFs by QRT-PCR (n = 4 per group). **E** The expression of CTGF in NRCFs by QRT-PCR (n = 4 per group). **F** The expression of Fn in NRCFs by QRT-PCR (n = 4 per group). **G** The expression of Col-I in NRCFs by QRT-PCR (n = 4 per group). **H** The expression of Col-III in NRCFs by QRT-PCR (n = 4 per group). **I** The expression of α-SMA in NRCFs by QRT-PCR (n = 4 per group). **J** Representative images of proteins by Western Blot. **K** The expression of PCNA in NRCFs by Western Blot (n = 4 per group). **L** The expression of CTGF in NRCFs by Western Blot (n = 4 per group). **M** The expression of Fn in NRCFs by Western Blot (n = 4 per group). **N** The expression of Col-I in NRCFs by Western Blot (n = 4 per group). **O** The expression of Col-III in NRCFs by Western Blot (n = 4 per group). **P** The expression of α-SMA in NRCFs by Western Blot (n = 4 per group). **Q** Representative images of α-SMA in NRCFs by immunofluorescence staining. Scale bar: 50 μm. **R** Quantification of immunofluorescence staining of α-SMA in NRCFs. NRCFs were starved for 24 h. Mig and Met were added ahead for 1h, then ISO was added for 24 h. Met was used as positive control. All genes were normalized to GAPDH. All proteins were normalized to β-Tubulin. The data were presented by Mean ± SEM, and analyzed using one-way ANOVA with Tukey’s post-hoc multiple comparison test. *, P < 0.05, **, P < 0.01, ***, P < 0.001, ****, P < 0.0001 vs. CON; #, P < 0.05, ##, P < 0.01, ###, P < 0.001, ####, P < 0.0001 vs. ISO

As mentioned above, we confirmed that Mig attenuated ISO-induced myocardial fibrosis in vivo. Since CFs activation is the central effector of cardiac fibrosis (Frangogiannis 2021), we then investigated the effect of Mig on CFs activation. To start with, we identified the isolation efficiency of NRCFs to ensure the purity (Fig. 3A). Morever, to exclude drug toxic impact in our experiments, we used CCK8 methods to choose 100 μM and 200 μM for further study (Fig. 3B). Cell proliferation, ECM deposition and transdifferentiation are three important aspects of fibroblast activation (Plikus et al. 2021). Expectedly, Mig markedly inhibited ISO-induced NRCFs proliferation (Fig. 3C). Meanwhile, cardiac fibrosis markers, such as CTGF and PCNA (markers of cell proliferation), Fn, Col-I and Col-III (markers of ECM stimulation) as well as α-SMA (a marker of cell transdifferentiation), were shown from these three aspects to identify the effect of Mig on ISO-induced CFs activation. Consistently, Mig reduced the expression of fibrotic markers both in mRNA (Fig. 3D–I) and protein levels (Fig. 3J–P). Furthermore, results of α-SMA fluorescence also proved the similar conclusion (Fig. 3Q, R). Taken together, these data indicated that Mig attenuated ISO-induced CFs activation.

The heart is mainly made up of cardiomyocytes and fibroblasts (Nuamnaichatia et al. 2018; Ottaviano et al. 2011). To clarify whether Mig alleviated ISO-induced cardiomyocyte apoptosis, we also investigated the effect of Mig on cardiomyocyte apoptosis. The data showed that Mig inhibited ISO-induced overexpression of Bax protein (a pro-apoptotic protein) (Figure S5A, B). In addition, Mig increased Bcl2 protein (an anti-apoptotic protein) level in NRCMs (Fig. S5A, C). In summary, Mig inhibited ISO-induced apoptosis of cardiomyocytes.

### Mig inhibits ISO-induced cardiac fibrosis by ERK, STAT3, Akt and GSK3β signaling

**Fig. 4:**
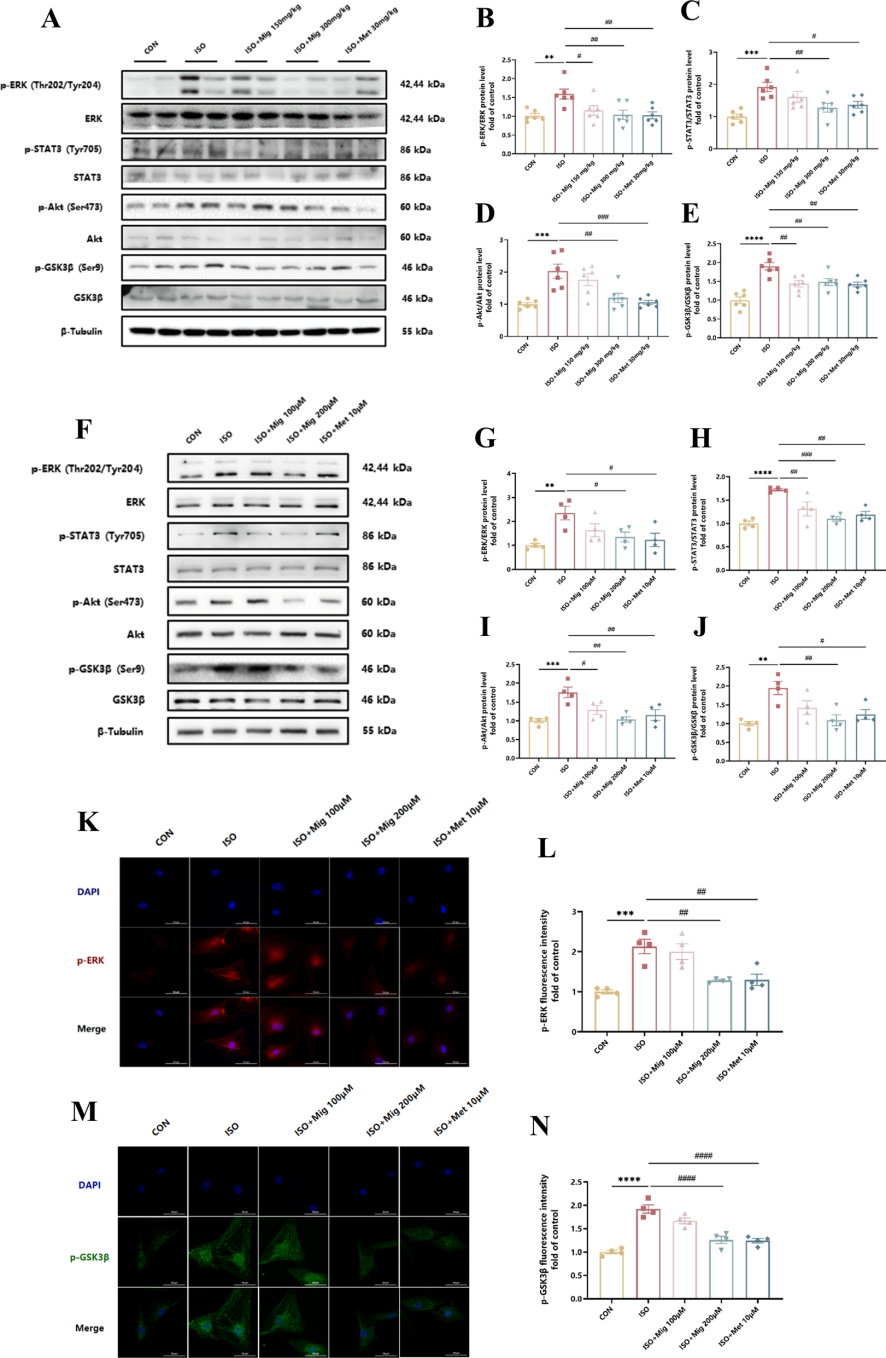
The mechanism of Mig on ISO-induced cardiac fibrosis. **A** Representative images of ERK, STAT3, Akt and GSK3β signal pathways in heart tissues by Western Blot. **B** The phosphorylation of ERK in heart tissues by Western Blot (n = 6 per group). **C** The phosphorylation of STAT3 in heart tissues by Western Blot (n = 6 per group). **D** The phosphorylation of Akt in heart tissues by Western Blot (n = 6 per group). **E** The phosphorylation of GSK3β in heart tissues by Western Blot (n = 6 per group). **F** Representative images of ERK, STAT3, Akt and GSK3β signal pathways in NRCFs by Western Blot. **G** The phosphorylation of ERK in NRCFs by Western Blot (n = 4 per group). **H** The phosphorylation of STAT3 in NRCFs by Western Blot (n = 4 per group). **I** The phosphorylation of Akt in NRCFs by Western Blot (n = 4 per group). **J** The phosphorylation of GSK3β in NRCFs by Western Blot (n = 4 per group). **K** Representative images of phosphorylated-ERK in NRCFs by immunofluorescence staining. Scale bar: 50 μm. **L** Quantification of immunofluorescence staining of phosphorylated-ERK in NRCFs (n = 4 per group). **M** Representative images of phosphorylated-GSK3β in NRCFs by immunofluorescence staining. Scale bar: 50 μm. **N** Quantification of immunofluorescence staining of phosphorylated-GSK3β in NRCFs (n = 4 per group). The ratio of phosphorylated protein to total protein reflects the activation of signaling pathways. NRCFs were starved for 24 h. Mig and Met were added ahead for 1h, then ISO was added for 24 h. Met was used as positive control. All proteins were normalized to β-Tubulin. The data were presented by Mean ± SEM, and analyzed using one-way ANOVA with Tukey’s post-hoc multiple comparison test. **, P < 0.01, ***, P < 0.001, ****, P < 0.0001 vs. CON; #, P < 0.05, ##, P < 0.01, ###, P < 0.001, ####, P < 0.0001 vs. ISO

To gain insight into the mechanism of Mig on cardiac fibrosis, we further explored several classical signaling of β-AR. ERK, STAT3, Akt and GSK3β, were then selected to detect both in vivo and in vitro. As expected, Mig suppressed protein phosphorylation levels of ERK, STAT3, Akt and GSK3β in ISO group in heart tissues (Fig. 4A–E). Consistently, Mig reduced ISO-induced protein phosphorylation levels ERK, STAT3, Akt and GSK3β in NRCFs (Fig. 4F–J). Besides, immunofluorescence staining of p-ERK (Fig. 4K, L) and p-GSK3β (Fig. 4M, N) also proved the same results. In conclusion, these data suggested that Mig inhibited ISO-induced cardiac fibrosis by ERK, STAT3, Akt and GSK3β signaling pathways. Besides, in ISO-induced cardiac fibrosis model, ISO significant caused ERK activation in both fibroblasts and cardiomyocytes, while Mig suppressed ERK activation (Figure S4).

The master cytokine TGF-β mediates tissue fibrosis associated with TGF-β-Smad2/3 signaling, which mobilizes Smad2 and Smad3 transcription factors that control fibrosis by promoting gene expression (Khalil et al. 2017). We investigated the ISO-induced TGF-β-Smad2/3 signaling pathway and found that the TGF-β-Smad2/3 signaling pathway was activated in our mode. More importantly, we also found that Mig inhibited the phosphorylation of Smad2/3 in cardiac fibroblasts and heart tissues (Figure S6).

### UGCG is enhanced in patients with heart failure and ISO-induced cardiac fibrosis both in vivo and in vitro

**Fig. 5:**
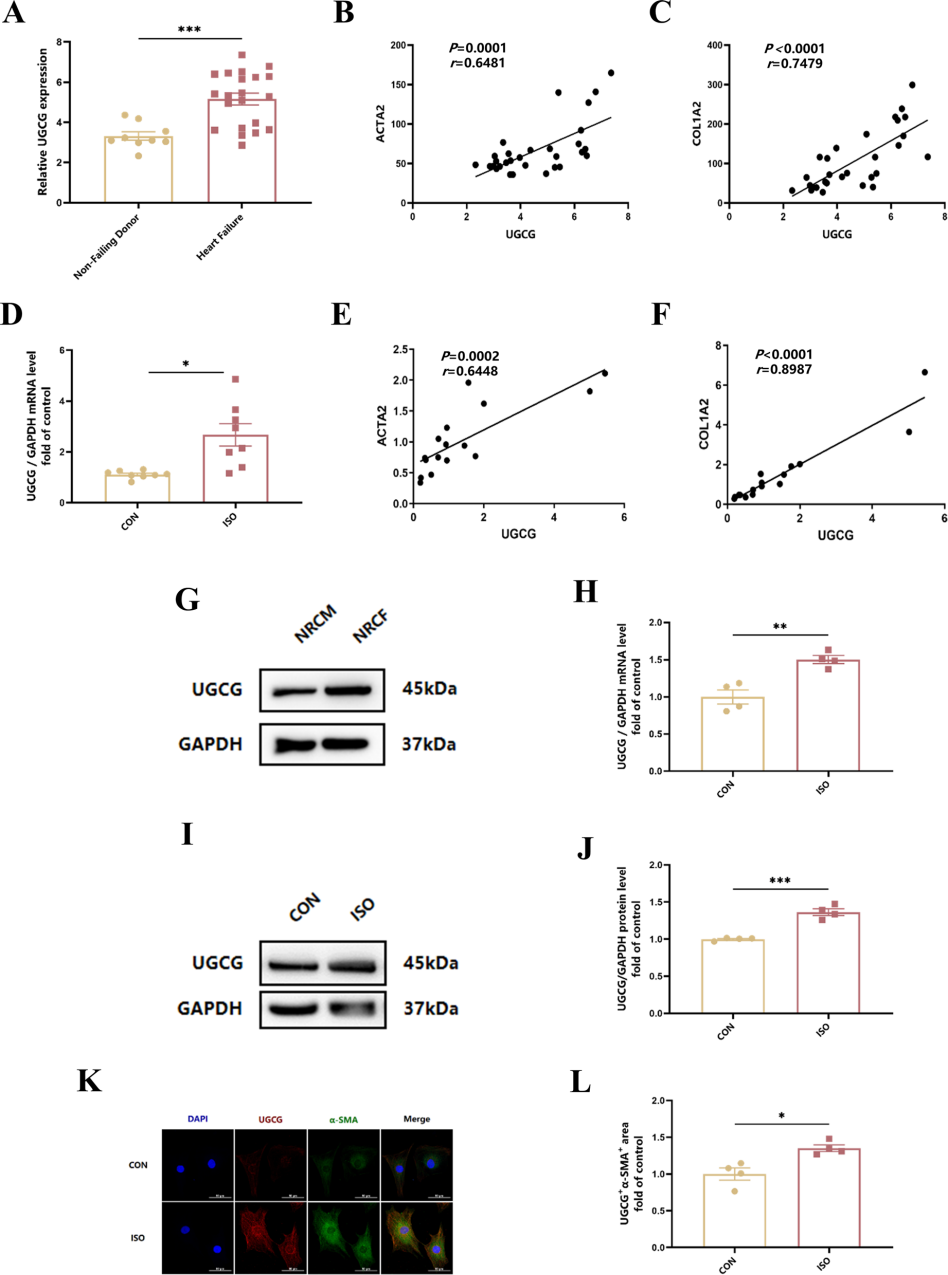
The expression of UGCG in patients with heart failure and ISO-induced mice cardiac fibrosis. **A** Relative mRNA level of UGCG in heart tissues in GSE135055. GEO2R analysis of UGCG in heart samples of patients with heart failure (n = 9) compared with healthy controls (n = 21). **B** The correlation between UGCG mRNA levels and those of ACTA2 in heart tissues. **C** The correlation between UGCG mRNA levels and those of COL1A2 in heart tissues. The correlation coefficient and the two-tailed significance are shown. **D** The expression of UGCG in heart tissues by QRT-PCR in mice (n = 8 per group). **E** The correlation between UGCG mRNA levels and those of ACTA2 in the same heart tissues (n = 8 per group). **F** The correlation between UGCG mRNA levels and those of COL1A2 in the same heart tissues (n = 8 per group). **G** Representative images of UGCG in NRCM and NRCF by Western Blot. **H** The expression of UGCG by QRT-PCR in NRCFs (n = 6 per group). **I** Representative images of UGCG by Western Blot in NRCFs. **J** Quantification of UGCG in NRCFs by Western Blot (n = 4 per group). **K** Representative images of UGCG and α-SMA in NRCFs by immunofluorescence staining. NRCFs were starved for 24 h, then ISO was added for 24h. **L** Quantification of UGCG^+^α-SMA^+^ area in ISO-induced NRCFs (n = 4 per group). Quantification of UGCG mRNA and protein levels were normalized to GAPDH. The data were presented by Mean ± SEM, and analyzed using two-tailed Student’s t-test. *, P < 0.05, **, P < 0.01, ***, P < 0.001 vs. Non-Failing Donor/CON

Since Mig is a classical inhibitor of UGCG, our data have demonstrated that Mig inhibited UGCG in ISO-induced cardiac fibrosis model in vivo (Figure S7). To further explore the underlying mechanism by which Mig ameliorates cardiac fibrosis, we then considered whether there is an intrinsic connection between UGCG and Mig on cardiac fibrosis. To clarify the function of UGCG in the development of HF, we tested UGCG relative mRNA level in a public GEO Dataset. In GSE135055, we found that the mRNA expression of UGCG was evidently higher in HF patients than healthy donor (Fig. 5A). To determine whether the expression of UGCG is associated with cardiac fibrosis, we proved UGCG had the correlation with ACTA2 and COL1A2, which are two biomarkers of cardiac fibrosis (Fig. 5B, C). Consistently, similar results were found in ISO-induced cardiac fibrosis mice (Figure S1 and Fig. 5D–F). However, little is known of UGCG in CFs. Therefore, we then proved that UGCG is expressed in NRCFs (Fig. 5G). We demonstrated that the increase of UGCG was mainly originated from cardiac fibroblasts rather than cardiomyocytes in heart tissues (Figure S3). We further explored whether UGCG expression changed with ISO treatment. Interestingly, the expression of UGCG was enhanced in both mRNA and protein levels (Fig. 5H–J). Moreover, the fluorescence of UGCG and α-SMA also demonstrated the same results (Fig. 5K). In summary, these data demonstrated that UGCG is enhanced in patients with HF and ISO-induced cardiac fibrosis.

### Mig ameliorates ISO-induced cardiac fibrosis via targeting UGCG in vitro

**Fig. 6:**
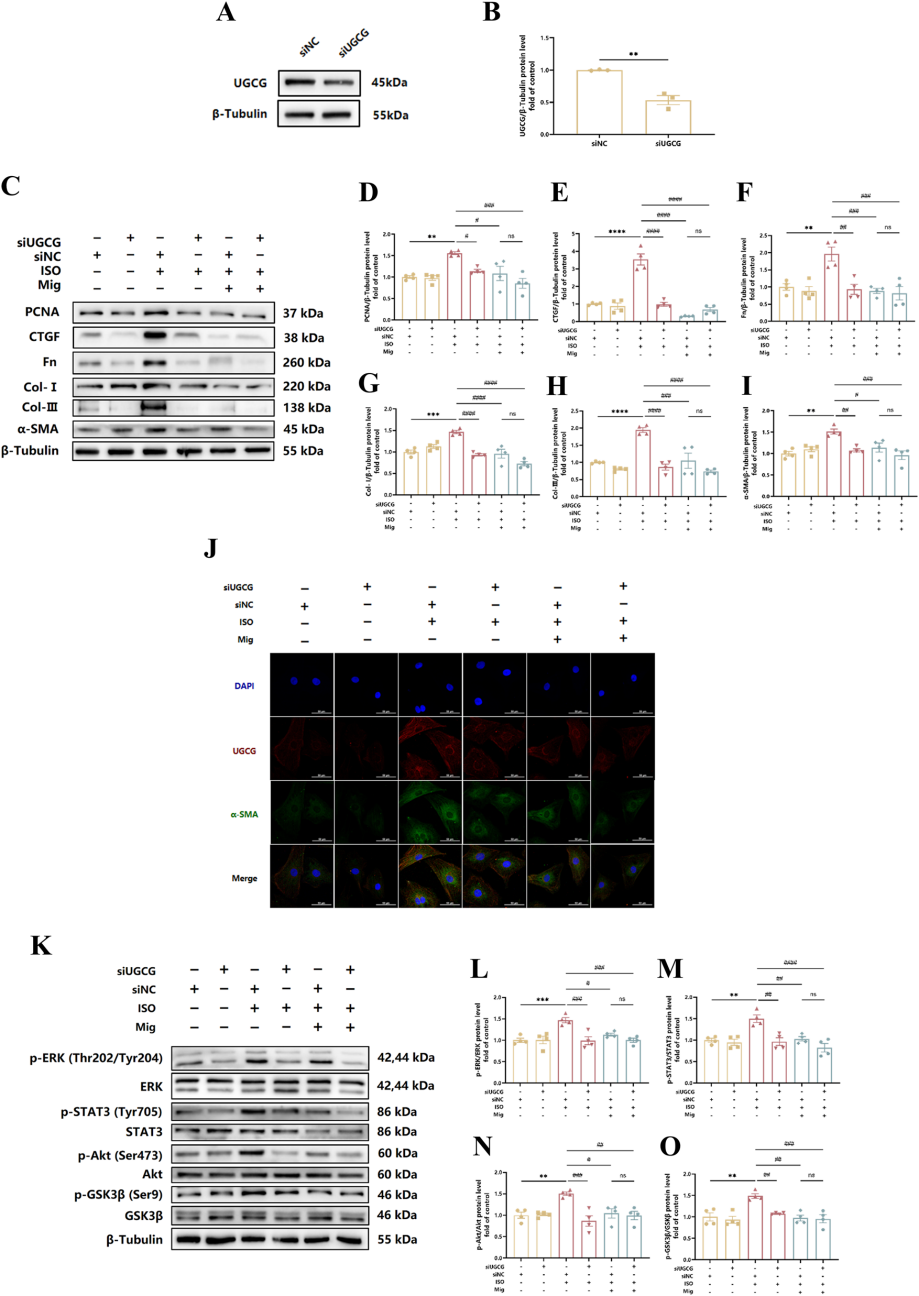
The mechanism of Mig on cardiac fibrosis via UGCG. **A** Representative images of UGCG knock down in NRCFs. **B** Quantification of UGCG knock down on protein level in NRCFs (n = 3 per group). **C** Representative images of Western Blot for PCNA, CTGF, Fn, Col-I, Col-III and α-SMA on Mig during UGCG knock down in NRCFs. **D** Quantification of PCNA on protein level in NRCFs (n = 4 per group). **E** Quantification of CTGF on protein level in NRCFs (n = 4 per group). **F** Quantification of Fn on protein level in NRCFs (n = 4 per group). **G** Quantification of Col-I on protein level in NRCFs (n = 4 per group). **H** Quantification of Col-III on protein level in NRCFs (n = 4 per group). **I** Quantification of α-SMA on protein level in NRCFs (n = 4 per group). **J** Representative images of immunofluorescence staining of UGCG and α-SMA in NRCFs. Scale bar: 50 μm. **K** Representative images of ERK, STAT3, Akt and GSK-3β signal pathways on Mig during UGCG knock down in NRCFs. **L** Quantification of ERK signal pathway on Mig during UGCG knock down in NRCFs (n = 4 per group). **M** Quantification of STAT3 signal pathways on Mig during UGCG knock down in NRCFs (n = 4 per group). **N** Quantification of Akt signal pathways on Mig during UGCG knock down in NRCFs (n = 4 per group). **O** Quan-tification of GSK-3β signal pathways on Mig during UGCG knock down in NRCFs (n = 4 per group). NRCFs were starved for 24 h. Mig and Met were added ahead for 1h, then ISO was added for 24 h. The protein levels of PCNA, CTGF, Fn, Col-I, Col-III and α-SMA were normalized to β-Tubulin. The data were shown as mean ± SEM (one-way ANOVA with Tukey’s post-hoc multiple comparison tests). **, P < 0.01, ***, P < 0.001 vs. siNC; #, P < 0.05, #, P < 0.05, ##, P < 0.01, ###, P < 0.001 vs. siUGCG + ISO; $, P < 0.05 vs. siNC + Mig + ISO

Since UGCG might had relationship with sympathetic activation-induced cardiac fibrosis, to further confirm whether the protective effect of Mig on cardiac fibrosis depended on UGCG, we then knocked down UGCG in NRCFs (Fig. 6A, B). Interestingly, Mig could not attenuated CFs activation indicators during UGCG knock down, including PCNA and CTGF (marker of cell proliferation), Fn, Col-I and Col-III (marker of ECM stimulation) as well as α-SMA (marker of cell transdifferentiation), while compared with NC knock down with Mig (Fig. 6C–I). In addition, immunofluorescence staining of UGCG and α-SMA also suggested similar conclusion (Fig. 6J). Additionally, mechanism research proved that Mig might not attenuated CFs activation downstream signaling, including ERK, STAT3, Akt and GSK3β, while compared with NC knock down with Mig (Fig. 6K–O). In conclusion, these data demonstrated that Mig attenuated ISO-induced cardiac fibrosis via targeting UGCG.

## Discussion

**Fig. 7:**
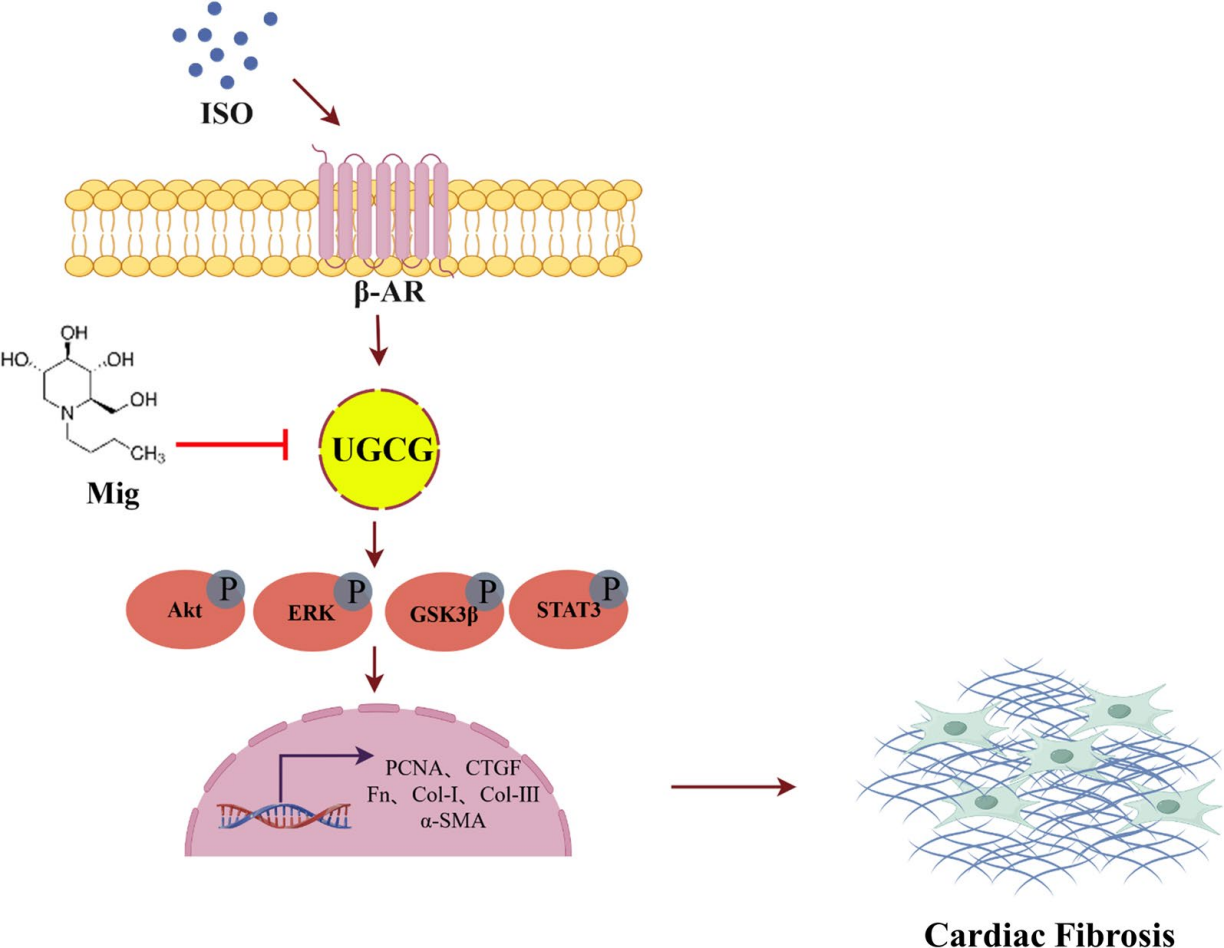
Working model of Mig on ISO-induced cardiac fibrosis

To begin with, our study found that Mig alleviated ISO-induced cardiac dysfunction and cardiac fibrosis in mice. Meanwhile, Mig also attenuated ISO-induced CFs activation. Besides, mechanism research showed that Mig could function in cardiac fibrosis by ERK, STAT3, Akt and GSK3β signalings both in vivo and in vitro. In addition, Mig played a role in cardiac fibrosis in a UGCG-dependent manner. In summary, these findings indicated that Miglustat ameliorated isoproterenol-induced cardiac fibrosis via targeting UGCG and UGCG might be a potential target for cardiac fibrosis (Fig. 7).

Under pathological conditions of heart, to increase cardiac output in the heart, β-adrenergic receptors (β-ARs) are activated to improve myocardial contractility and sympathetic activity increased (Rockman HA et al. 2002). However, long-term stimulation of β-ARs can lead to other pathologic cardiac dysfunction, such as cardiac fibrosis (Frangogiannis NG 2021), apoptosis (Zaugg et al. 2000), and even heart failure (Lohse et al. 2003). The β-ARs is a typical member of G protein-coupled receptor (GPCR), which mainly plays a role in regulating the activity of cardiovascular system and is the most richly expressed receptor in the heart. The abnormality of β-AR signaling system is the basis for the occurrence and development of many cardiovascular diseases (Carter et al. 2015; Cohn et al. 1990). When ligands bind to β-AR receptors, β-ARs downstream signaling pathways are activated, including PKA/cAMP and PI3K/Akt signaling pathways, causing changes in enzyme activity, ion channel activity and transcription factor activity, thereby regulating cell growth and metabolism, cytoskeletal structure and gene expression (Nuamnaichati et al. 2018; Spadari et al. 2017). In addition, β-AR can also transmit signals through G protein independent pathways, including ERK and Src (Dostal et al. 2015; Wei et al. 2024; Li et al. 2023). The animal model used in this study was an ISO-induced cardiac fibrosis model based on β-AR overactivation. And the positive drug used in this model is metoprolol (Met), which is a classic β-blocker. It competitively inhibited the β-AR, prevented sympathetic overactivation from damaging the heart, and improved ventricular and blood vessel remodeling and function. And, Met has been shown to be beneficial in improving cardiac fibrosis in patients with heart failure (Nuamnaichati et al. 2018).

We further evaluated the efficacy of Mig in a cardiac fibrosis model with β-AR overactivation, and our results showed that Mig was able to alleviate ISO-induced cardiac fibroblast activation and cardiac fibrosis. In previous studies, Mig was associated with a mass of fibrotic diseases, involving pulmonary fibrosis and liver fibrosis (Nakamura et al. 2023; Iwanaga et al. 2023). As mentioned above, TGF-β receptor and its downstream signaling are the most classical signaling in tissue fibrosis (Plikus et al. 2021; Frangogiannis et al. 2020; Zhao et al. 2022). Other mechanism studies have shown that the impact of Mig connected with TGF-β/Smad signaling. As for pulmonary fibrosis, Mig ameliorated pulmonary fibrosis in a BLM-induced lung injury model by inhibiting TGF-β1-induced Smad2/3 nuclear translocation, rather than Smad2/3 phosphorylation (Nakamura et al. 2023). Because Smad2/3 phosphorylation was not altered, it was speculated that Mig did not affect the phosphorylation of TGF-β type I receptor during this process. As for liver fibrosis, results indicated that Mig alleviated CCl4-induced liver fibrosis by inhibiting the phosphorylation of Smad2/3 induced by hepatic stellate cells. However, the phosphorylation of TGF-β type I receptor was also inhibited during this process (Iwanaga et al. 2023). These findings suggested that the suppressed effect of Mig in liver fibrosis relied on the phosphorylation of TGF-β type I receptor. According to the different mechanisms of Mig in different fibrotic diseases, we ulteriorly explore the mechanism of Mig in myocardial fibrosis. Excessive activation of β-AR and its downstream signaling pathways play a central regulatory role in sympathetic stress-induced cardiac fibrosis (Cao et al. 2021; Fu et al. 2012), which we are concerned about. Mig can inhibit the classic β-AR downstream signaling pathway and pro-fibrotic factors through UGCG to play an anti-fibrotic role. At the same time, we also detected the effect of Mig on TGF-β signaling pathway, and the results showed that Mig could also inhibit TGF-β downstream signaling pathway. In these studies, we noted that the ERK signaling pathway is also involved in TGF-β1-mediated cardiac remodeling and cardiac fibrosis (Garlapati et al. 2023, 2024; Gao et al. 2009). Therefore, we speculated that in sympathetic stress-induced cardiac fibrosis, the downstream β-AR signaling pathway and the downstream TGF-β signaling pathway may have a cross-talk effect, and its internal mechanism needs to be further studied. In conclusion, in the sympathetic stress-induced cardiac fibrosis we studied, Mig has a regulatory effect on both β-AR and TGF-β downstream key signals.

Meanwhile, our results demonstrated that the effect of Mig on cardiac fibrosis is dependent on its target UGCG. To start with, UGCG plays a key role in the heart, especially in cardiomyocytes. Cardiac hypertrophy is one of the main pathological manifestations of cardiomyocytes, and cardiac β-AR plays a key role in maintaining cardiac function and regulating cardiac remodeling (Nakamura 2018; Ali et al. 2019). On the one hand, UGCG has shown to directly regulate β-AR in the heart. Knock down UGCG alleviated the β1-AR internalization and transport in heart, and aggravated cardiac hypertrophy caused by sympathetic overactivation and cardiac function (Andersson et al. 2021). On the other hand, UGCG also indirectly affected by the downstream signaling pathway of β-AR through synergistic interactions with other molecules. Furthermore, studies have reported that UGCG regulated cardiac hypertrophy through connection with Beta-1,4-galactosyltransferase 5 (B4GalT5) to mediate β-AR-regulated mitochondrial oxidative stress and ERK signaling pathway (Cui et al. 2023). In addition, targeting UGCG to treat cardiac hypertrophy in chronic kidney disease has also been reported (Baccam et al. 2022). In our animal model, we thought that cardiac fibroblasts activation rather than cardiomyocytes apoptosis, was the main pathological process at the end stage of cardiac fibrosis. Meanwhile, we also found that Miglustat had protective effects on cardiomyocytes. However, whether Miglustat functioned on cardiomyocytes apoptosis by targeting UGCG or not, was still remained to be explored. We also found the function and mechanism of UGCG in cardiac fibroblasts for the first time and demonstrated that Mig reduced cardiac fibrosis caused by sympathetic overactivation, and this process depends on UGCG. Further studies are still need to explore whether UGCG directly regulates β-AR to mediate cardiac fibrosis.

## Conclusion

In conclusion, our study demonstrated that Miglustat ameliorated isoproterenol-induced cardiac fibrosis via targeting UGCG and UGCG might be a potential target for cardiac fibrosis.

## Supplementary Information

Additional file 1.

## Data Availability

No datasets were generated or analysed during the current study.

## Abbreviations

β-AR: β-Adrenergic receptor
Mig: Miglustat
ISO: Isoproterenol
UGCG: UDP-glucose ceramide glycosyltransferase
B4GalT5: Beta-1,4-galactosyltransferase 5
HF: Heart failure
GSLs: Glycosphingolipids
Met: Metoprolol
CON: Control
cTnT: Cardiac Troponin T
LVPW;d: Left ventricular posterior wall thickness
LVEDV: Left Ventricular End Diastolic Volume
LVESV: Left Ventricular End Systolic Volume
LVEF: Left ventriclular ejection fraction
LVFS: Left ventricular fractional shortening
NRCFs: Neonatal rat cardiac fibroblasts
NRCMs: Neonatal rat cardiomyocytes
DMEM: Dulbecco’s modified Eagle medium
QRT-PCR: Quantitative Real-time PCR
Col-I: Collagen I
Col-III: Collagen III
Fn: Fibronectin
CTGF: Connective Tissue Growth Factor
PCNA: Proliferating Cell Nuclear Antigen
α-SMA: Alpha Smooth Muscle Actin
ANP: Atrial Natriuretic Peptide
BNP: Brain Natriuretic Peptide
HW/BW: The ratio of heart weight to body weight
HW/TL: The ratio of heart weight to tibia length
CFs: Cardiac fibroblasts
