# Correction: Miglustat ameliorates isoproterenol-induced cardiac fibrosis via targeting UGCG

**DOI:** 10.1186/s10020-025-01360-w

**Published:** 2025-09-23

**Authors:** Jing Liu, Wenqi Li, Ran Jiao, Zhigang Liu, Tiantian Zhang, Dan Chai, Lingxin Meng, Zhongyi Yang, Yuming Liu, Hongliang Wu, Xiaoting Gu, Xiaohe Li, Cheng Yang

**Affiliations:** 1 State Key Laboratory of Medicinal Chemical Biology, College of Pharmacy, Nankai University, Haihe Education Park, 38 Tongyan Road, Tianjin, 300353 China; 2 Department of Anesthesiology, National Clinical Research Center for Cancer/Cancer Hospital, National Cancer Center, Chinese Academy of Medical Sciences, Peking Union Medical College, No. 17 Nanli, Panjiayuan, Chaoyang District, Beijing, China; 3 Tianjin Key Laboratory of Molecular Drug Research, International Joint Academy of Biomedicine, Tianjin, 300457 China

**Correction: Mole Med 31, 55 (2025)**

**https://doi.org/10.1186/s10020-025-01093-w**

In this article [1], Fig. 2 panel B and Fig. 6 panel N were incorrect. For completeness and transparency, both correct and incorrect versions are displayed below.

The original article has been corrected.

Incorrect Fig. 2

**Figure:**
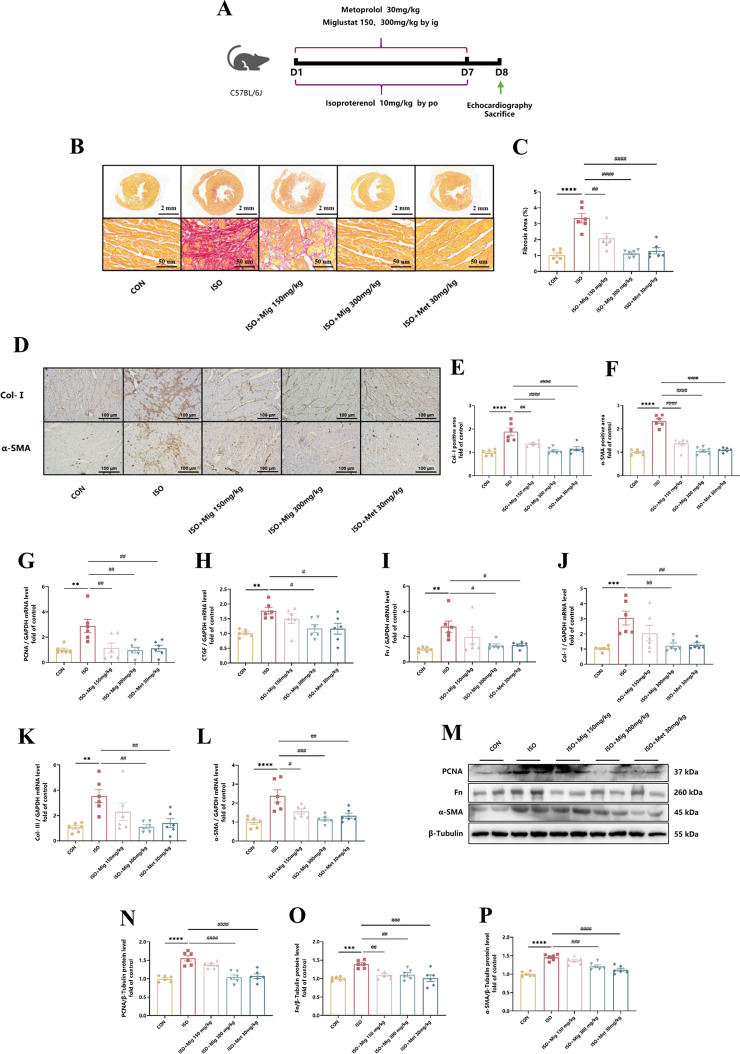
**Fig. 2** The effect of Mig on ISO-induced cardiac fibrosis. **A** Schematic representation of the animal model. **B** Representative 1 × and 20 × images of picrosirius red-stained in heart tissues. Scale bar (upper): 2 mm, scale bar (lower): 50 μm. **C** Quantification of picrosirius red-stained in heart tissues (*n* = 6 per group). **D** Representative 20 × images of Col-I and α-SMA immumohistochemical staining of heart tissues. Scale bar: 100 μm. **E** Quantification of Col-I immumohistochemical staining of heart tissues (*n* = 6 per group). **F** Quantification of α-SMA immumohistochemical staining of heart tissues (*n* = 6 per group). **G** The expression of PCNA in heart tissues by QRT-PCR (*n* = 6 per group). **H** The expression of CTGF in heart tissues by QRT-PCR (*n* = 6 per group). **I** The expression of Fn in heart tissues by QRT-PCR (*n* = 6 per group). **J** The expression of Col-I in heart tissues by QRT-PCR (*n* = 6 per group). **K** The expression of Col-III in heart tissues by QRT-PCR (*n* = 6 per group). **L** The expression of α-SMA in heart tissues by QRT-PCR (*n* = 6 per group). **M** Representative images of PCNA, Fn and α-SMA in heart tissues by Western Blot. **N** The expression of PCNA in heart tissues by Western Blot (*n* = 6 per group). **O** The expression of Fn in heart tissues by Western Blot (*n* = 6 per group). **P** The expression of α-SMA in heart tissues by Western Blot (*n* = 6 per group). Met was used as positive control. All the genes were normalized to GAPDH. The data were. presented by Mean ± SEM, and analyzed using one-way ANOVA with Tukey’s post-hoc multiple comparison test. **, *P* < 0.01, ***, *P* < 0.001, ****, *P* < 0.0001 vs. CON; #, *P* < 0.05, ##, *P* < 0.01, ###, *P* < 0.001, ####, *P* < 0.0001 vs. ISO

Correct Fig. 2

**Fig. 2:**
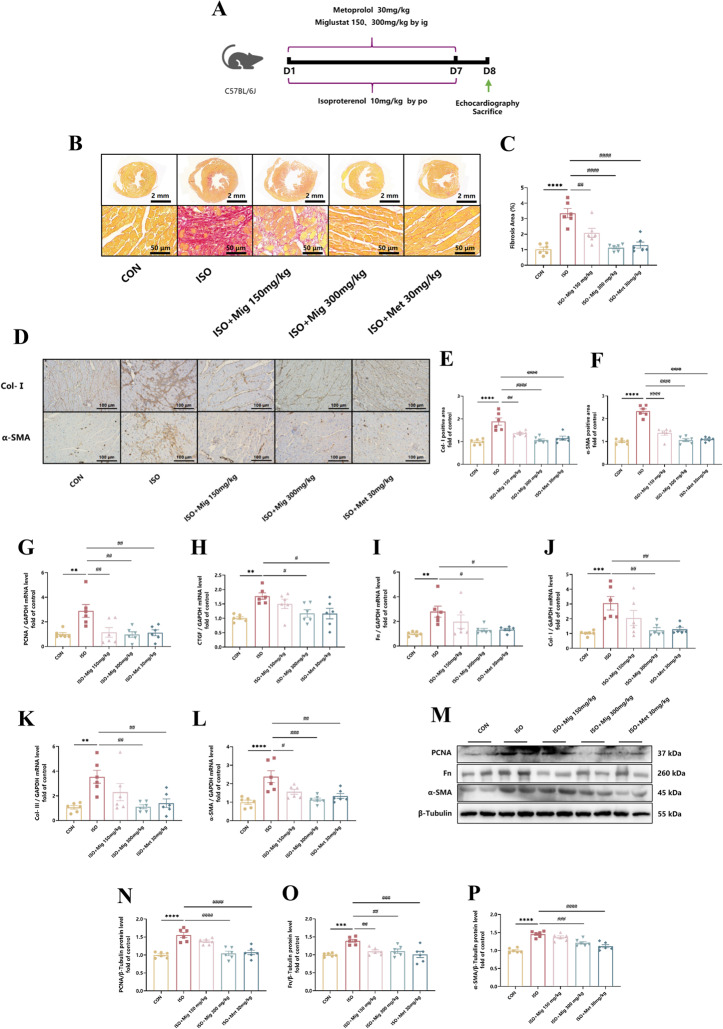
The effect of Mig on ISO-induced cardiac fibrosis. **A** Schematic representation of the animal model. **B** Representative 1 × and 20 × images of picrosirius red-stained in heart tissues. Scale bar (upper): 2 mm, scale bar (lower): 50 μm. **C** Quantification of picrosirius red-stained in heart tissues (*n* = 6 per group). **D** Representative 20 × images of Col-I and α-SMA immumohistochemical staining of heart tissues. Scale bar: 100 μm. **E** Quantification of Col-I immumohistochemical staining of heart tissues (*n* = 6 per group). **F** Quantification of α-SMA immumohistochemical staining of heart tissues (*n* = 6 per group). **G** The expression of PCNA in heart tissues by QRT-PCR (*n* = 6 per group). **H** The expression of CTGF in heart tissues by QRT-PCR (*n* = 6 per group). **I** The expression of Fn in heart tissues by QRT-PCR (*n* = 6 per group). **J** The expression of Col-I in heart tissues by QRT-PCR (*n* = 6 per group). **K** The expression of Col-III in heart tissues by QRT-PCR (*n* = 6 per group). **L** The expression of α-SMA in heart tissues by QRT-PCR (*n* = 6 per group). **M** Representative images of PCNA, Fn and α-SMA in heart tissues by Western Blot. **N** The expression of PCNA in heart tissues by Western Blot (*n* = 6 per group). O The expression of Fn in heart tissues by Western Blot (*n* = 6 per group). **P** The expression of α-SMA in heart tissues by Western Blot (*n* = 6 per group). Met was used as positive control. All the genes were normalized to GAPDH. The data were. presented by Mean ± SEM, and analyzed using one-way ANOVA with Tukey’s post-hoc multiple comparison test. **, *P* < 0.01, ***, *P* < 0.001, ****, *P* < 0.0001 vs. CON; #, *P* < 0.05, ##, *P* < 0.01, ###, *P* < 0.001, ####, *P* < 0.0001 vs. ISO

Incorrect Fig. 6

**Figure:**
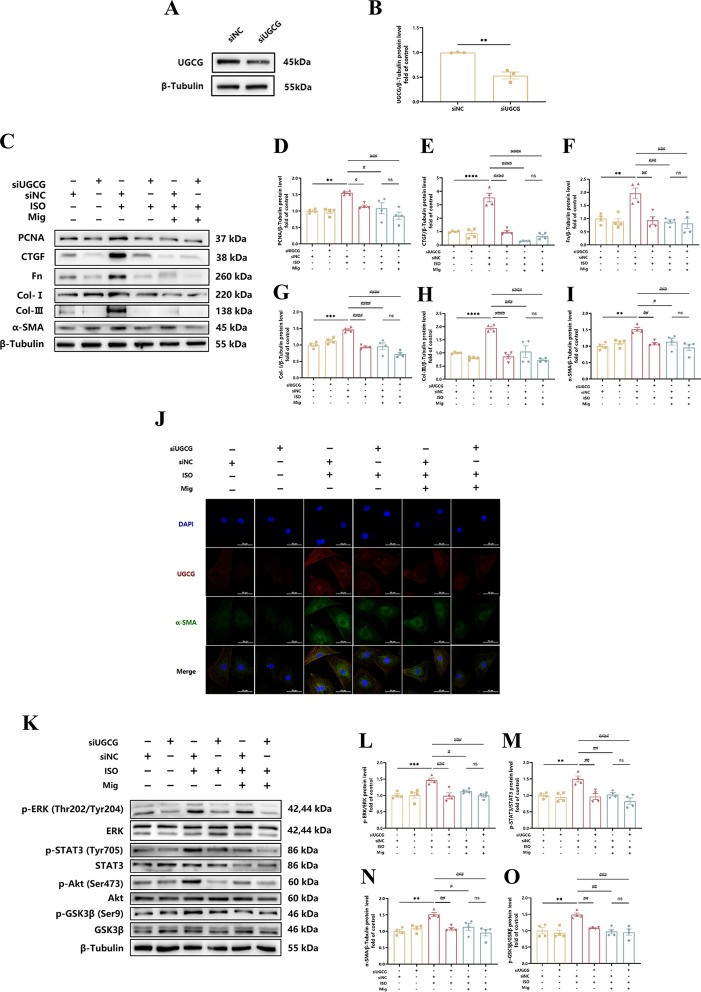
**Fig. 6** The mechanism of Mig on cardiac fibrosis via UGCG. **A** Representative images of UGCG knock down in NRCFs. **B** Quantification of UGCG knock down on protein level in NRCFs (*n* = 3 per group). **C** Representative images of Western Blot for PCNA, CTGF, Fn, Col-I, Col-III and α-SMA on Mig during UGCG knock down in NRCFs. **D** Quantification of PCNA on protein level in NRCFs (*n* = 4 per group). **E** Quantification of CTGF on protein level in NRCFs (*n* = 4 per group). **F** Quantification of Fn on protein level in NRCFs (*n* = 4 per group). G Quantification of Col-I on protein level in NRCFs (*n* = 4 per group). **H** Quantification of Col-III on protein level in NRCFs (*n* = 4 per group). **I** Quantification of α-SMA on protein level in NRCFs (*n* = 4 per group). **J** Representative images of immunofluorescence staining of UGCG and α-SMA in NRCFs. Scale bar: 50 μm. **K** Representative images of ERK, STAT3, Akt and GSK-3β signal pathways on Mig during UGCG knock down in NRCFs. **L** Quantification of ERK signal pathway on Mig during UGCG knock down in NRCFs (*n* = 4 per group). **M** Quantification of STAT3 signal pathways on Mig during UGCG knock down in NRCFs (*n* = 4 per group). **N** Quantification of Akt signal pathways on Mig during UGCG knock down in NRCFs (*n* = 4 per group). **O** Quan-tification of GSK-3β signal pathways on Mig during UGCG knock down in NRCFs (*n* = 4 per group). NRCFs were starved for 24 h. Mig and Met were added ahead for 1 h, then ISO was added for 24 h. The protein levels of PCNA, CTGF, Fn, Col-I, Col-III and α-SMA were normalized to β-Tubulin. The data were shown as mean ± SEM (one-way ANOVA with Tukey’s post-hoc multiple comparison tests). **, *P* < 0.01, ***, *P* < 0.001 vs. siNC; #, *P* < 0.05, #, *P* < 0.05, ##, *P* < 0.01, ###, *P* < 0.001 vs. siUGCG + ISO; $, *P* < 0.05 vs. siNC + Mig + ISO

Correct Fig. 6.

**Fig. 6:**
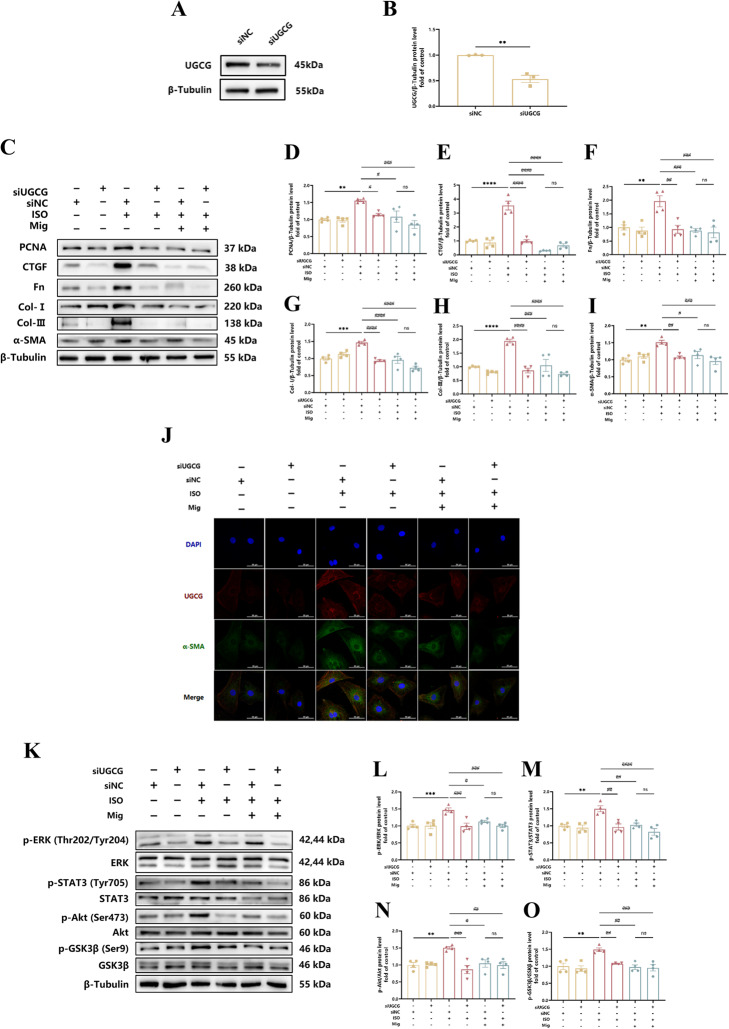
The mechanism of Mig on cardiac fibrosis via UGCG. **A** Representative images of UGCG knock down in NRCFs. **B** Quantification of UGCG knock down on protein level in NRCFs (*n* = 3 per group). **C** Representative images of Western Blot for PCNA, CTGF, Fn, Col-I, Col-III and α-SMA on Mig during UGCG knock down in NRCFs. **D** Quantification of PCNA on protein level in NRCFs (*n* = 4 per group). **E** Quantification of CTGF on protein level in NRCFs (*n* = 4 per group). **F** Quantification of Fn on protein level in NRCFs (*n* = 4 per group). **G** Quantification of Col-I on protein level in NRCFs (*n* = 4 per group). **H** Quantification of Col-III on protein level in NRCFs (*n* = 4 per group). **I** Quantification of α-SMA on protein level in NRCFs (*n* = 4 per group). **J** Representative images of immunofluorescence staining of UGCG and α-SMA in NRCFs. Scale bar: 50 μm. **K** Representative images of ERK, STAT3, Akt and GSK-3β signal pathways on Mig during UGCG knock down in NRCFs. **L** Quantification of ERK signal pathway on Mig during UGCG knock down in NRCFs (*n* = 4 per group). **M** Quantification of STAT3 signal pathways on Mig during UGCG knock down in NRCFs (*n* = 4 per group). **N** Quantification of Akt signal pathways on Mig during UGCG knock down in NRCFs (*n* = 4 per group). **O** Quan-tification of GSK-3β signal pathways on Mig during UGCG knock down in NRCFs (*n* = 4 per group). NRCFs were starved for 24 h. Mig and Met were added ahead for 1 h, then ISO was added for 24 h. The protein levels of PCNA, CTGF, Fn, Col-I, Col-III and α-SMA were normalized to β-Tubulin. The data were shown as mean ± SEM (one-way ANOVA with Tukey’s post-hoc multiple comparison tests). **, *P* < 0.01, ***, *P* < 0.001 vs. siNC; #, *P* < 0.05, #, *P* < 0.05, ##, *P* < 0.01, ###, *P* < 0.001 vs. siUGCG + ISO; $, *P* < 0.05 vs. siNC + Mig + ISO
