# Supplementary material for: Miglustat ameliorates isoproterenol-induced cardiac fibrosis via targeting UGCG
Source: Mol Med. 2025 Feb 11;31:55. doi: 10.1186/s10020-025-01093-w (PMC11812238; doi:10.1186/s10020-025-01093-w)
Supplement: Supplementary file 1 — Additional file 1. [file 10020_2025_1093_MOESM1_ESM.docx]

Supplementary Material

# Supplementary Figures and Tables

##
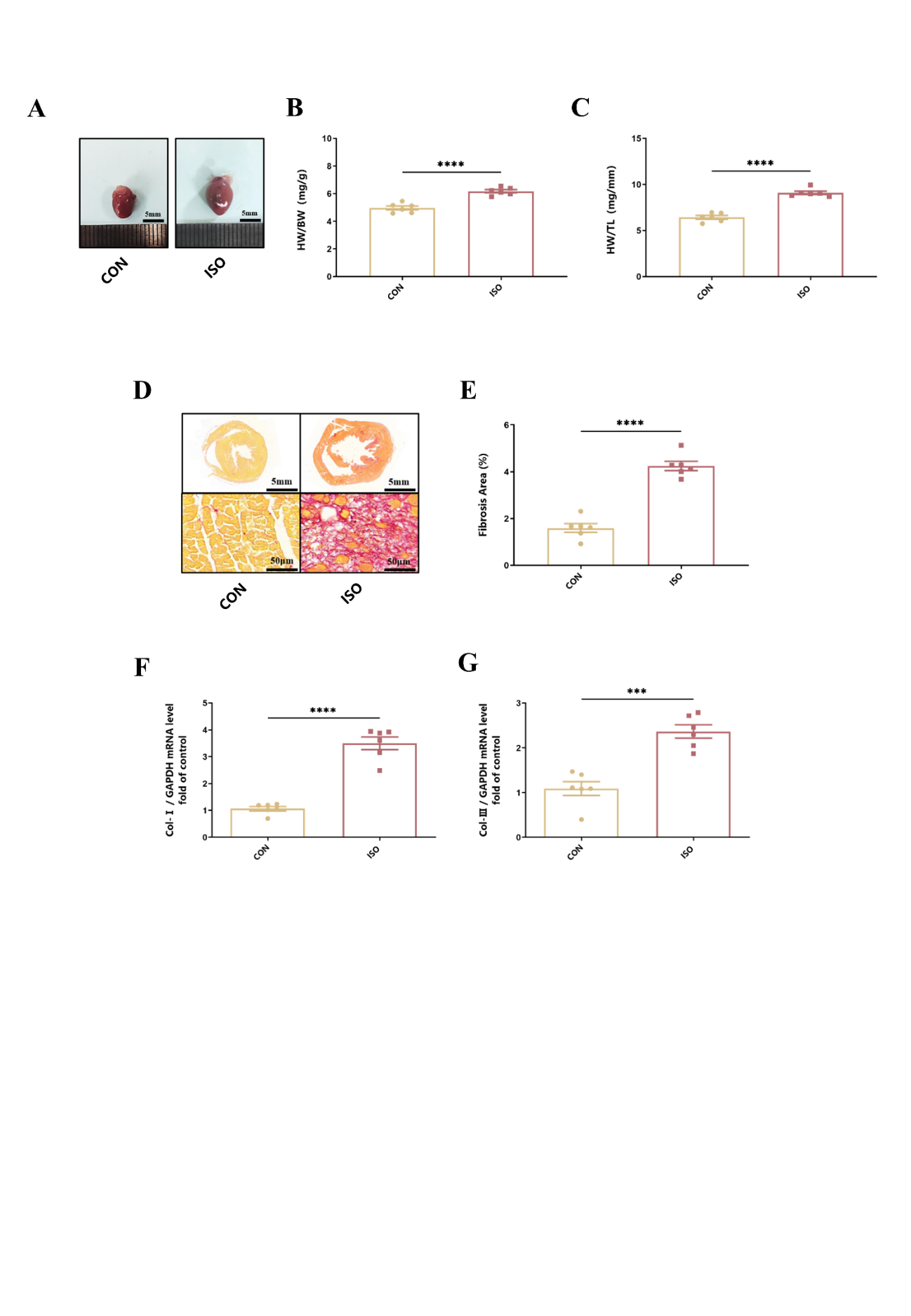
Supplementary Figures

**Figure S1.** **The establishment of ISO-induced cardiac fibrosis model**. (A) Representative images of heart size. Scale bar: 5 mm. (B) Quantitative analysis of HW/BW ratio (n=6 per group). (C) Quantitative analysis of HW/TL ratio (n=6 per group). (D) Representative 1× and 40× images of picrosirius red-stained in heart tissues. Scale bar (upper): 5 mm, scale bar (lower): 50 μm. (E) Quantification of picrosirius red-stained in heart tissues (n=6 per group). (F) The mRNA level of Col-Ⅰ in heart tissues (n=6 per group). (G) The mRNA level of Col-Ⅲ in heart tissues (n=6 per group). Quantification of Col-Ⅰ and Col-Ⅲ mRNA levels were normalized to GAPDH. The data were
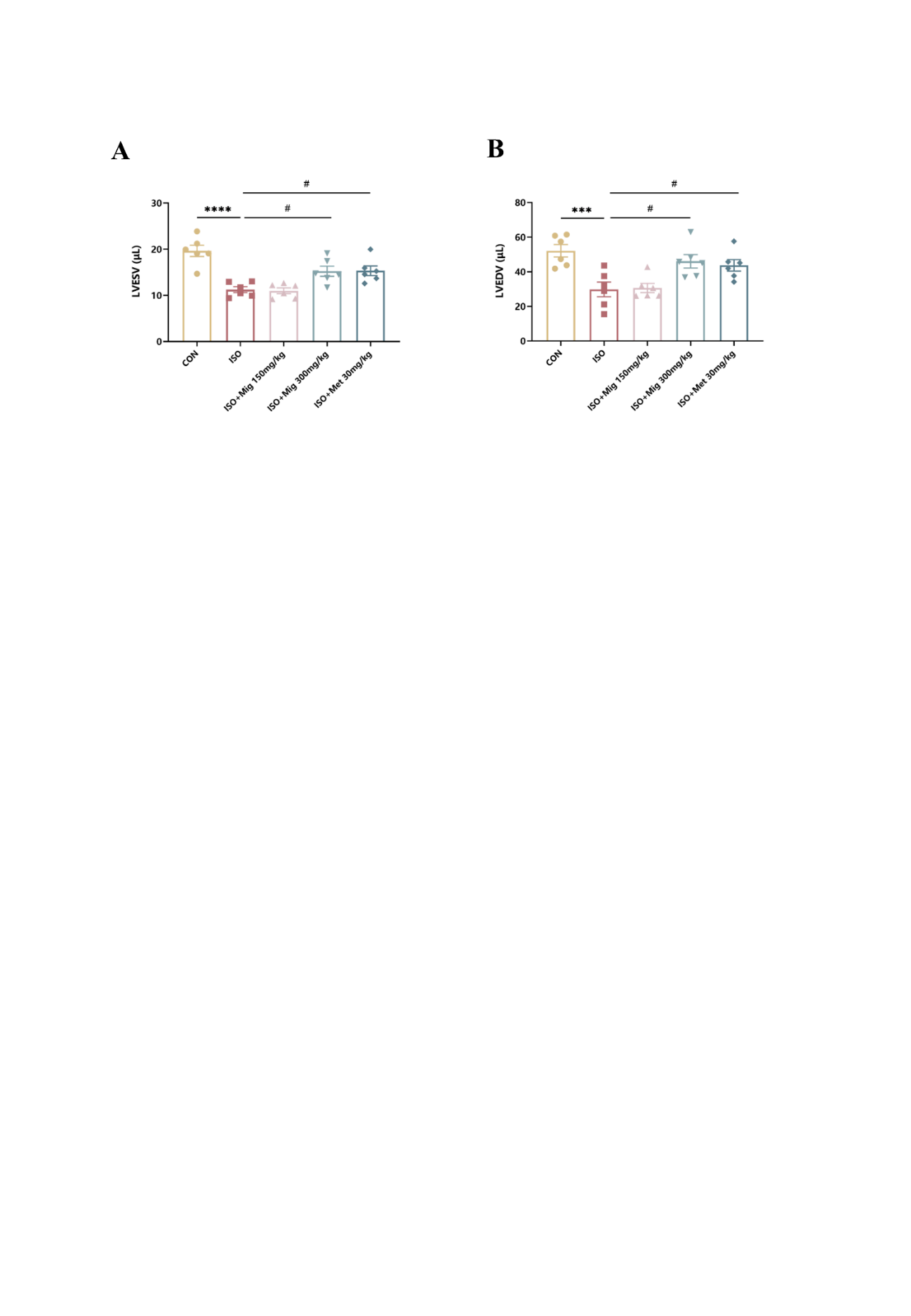
shown as Mean ± SEM. ***, P＜0.001, ****, P＜0.0001 vs. CON.


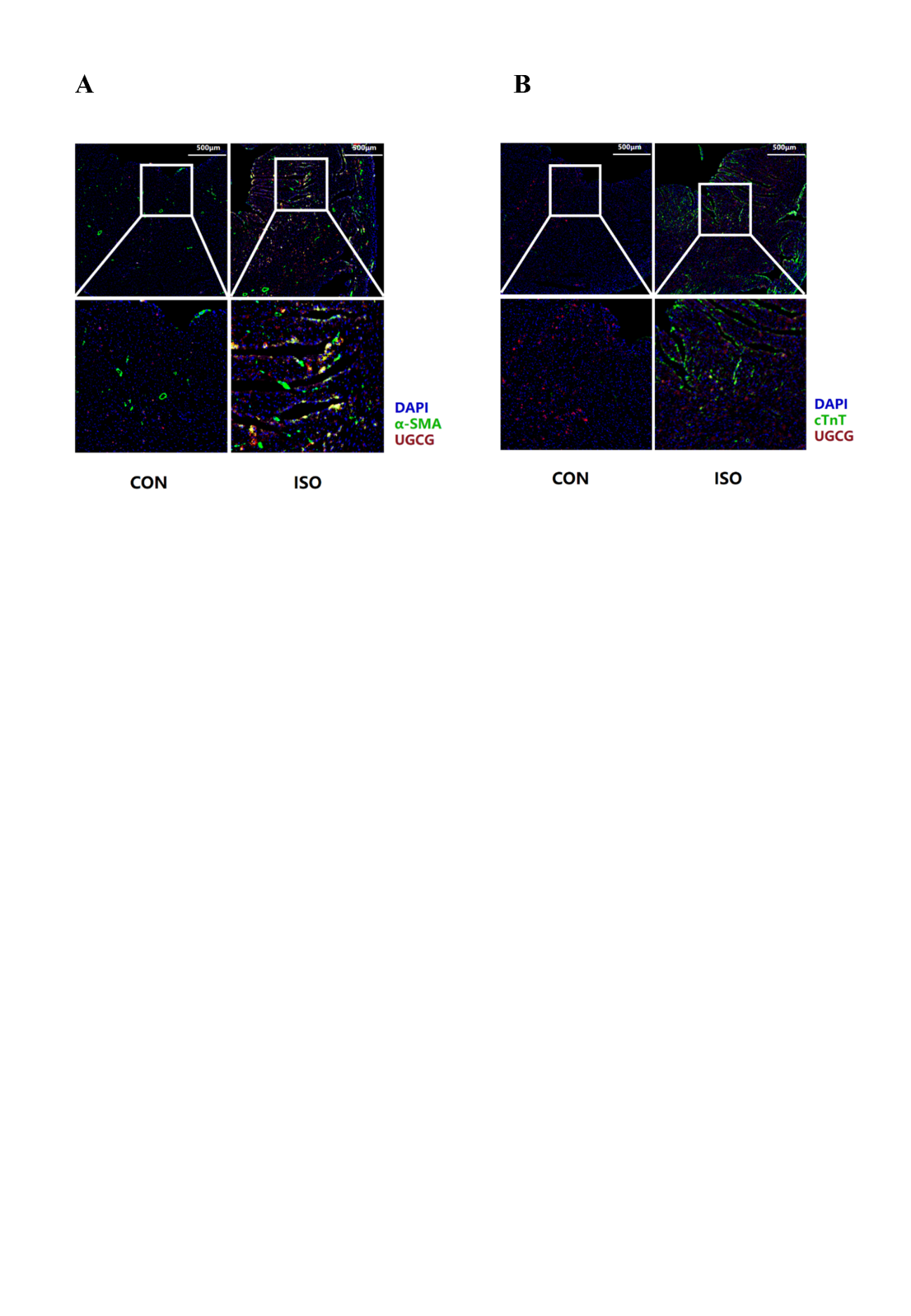
**Figure S2.** **Mig ameliorates ISO-induced cardiac dysfunction.**  (A) Echocardiographic measurement of LVESV(μL) (n=6 per group). (B) Echocardiographic measurement of LVEDV(μL) (n=6 per group). The data were shown as Mean ± SEM (one-way ANOVA with Tukey’s post-hoc multiple comparison tests). ***, P＜0.001, ****, P＜0.0001 vs. CON; #, P＜0.05 vs. ISO.

**Figure S3.** **The UGCG are mainly expressed in activated myofibroblasts in ISO-induced cardiac fibrosis.** (A) On day 8 after ISO induction, heart tissues were stained with antibodies against α-SMA and UGCG. Scale bar: 500 μm. (B) On day 8 after ISO induction, heart tissues were stained with antibodies against cTnT and UGCG. Scale bar: 500 μm.


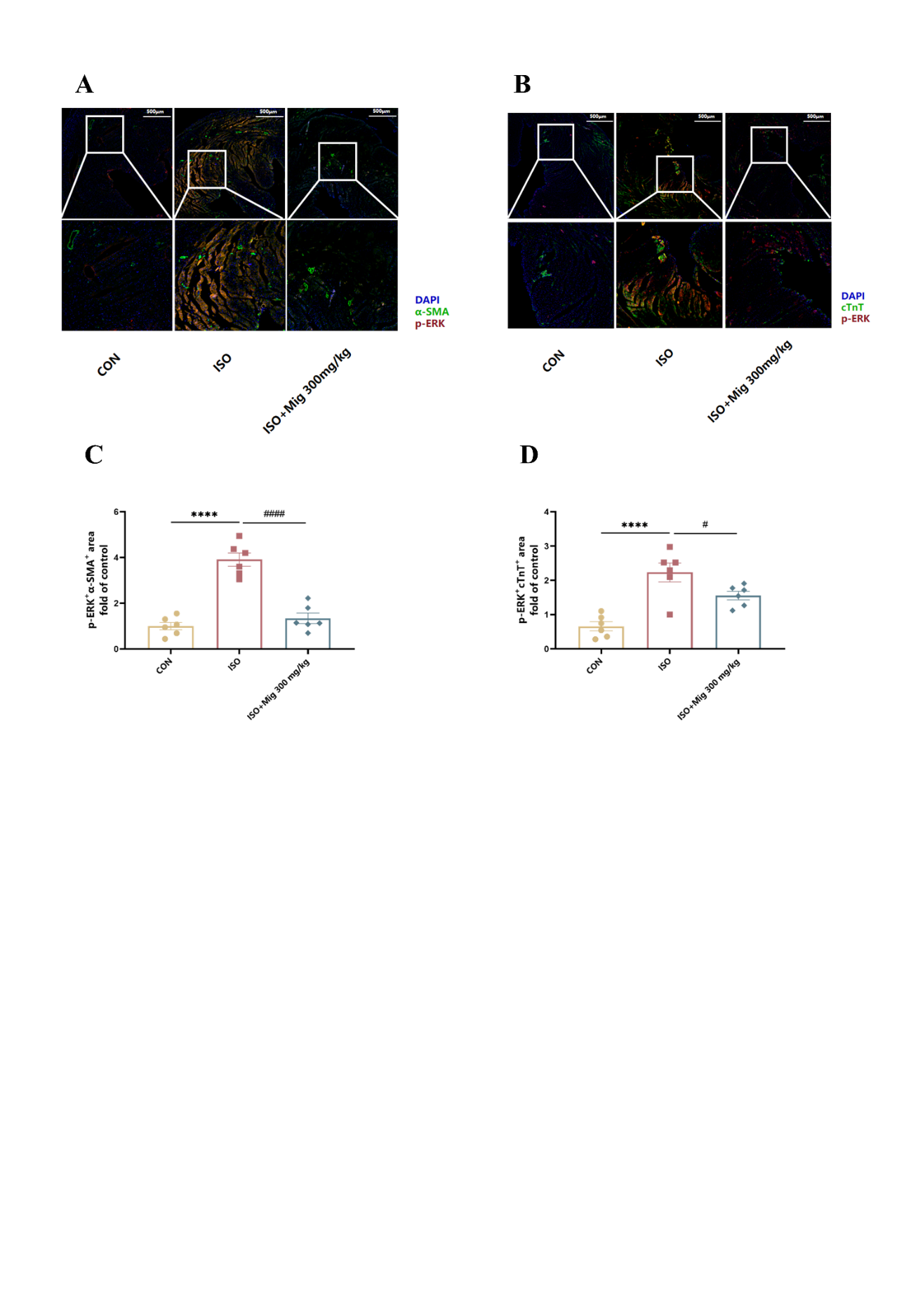


**Figure S4.** **Mig inhibits ERK activation in ISO-induced cardiac fibrosis.** (A) On day 0 and day 7 after ISO induction, heart tissues were stained with antibodies against α-SMA and p-ERK (n=6 per group). Scale bar: 500 μm. (B) On day 0 and day 7 after ISO induction, heart tissues were stained with antibodies against cTnT and p-ERK (n=6 per group). Scale bar: 500 μm. The data were shown as Mean ± SEM (one-way ANOVA with Tukey’s post-hoc multiple comparison tests). ****, P＜0.0001 vs. CON; #, P<0.05, ####, P＜0.0001 vs. ISO.


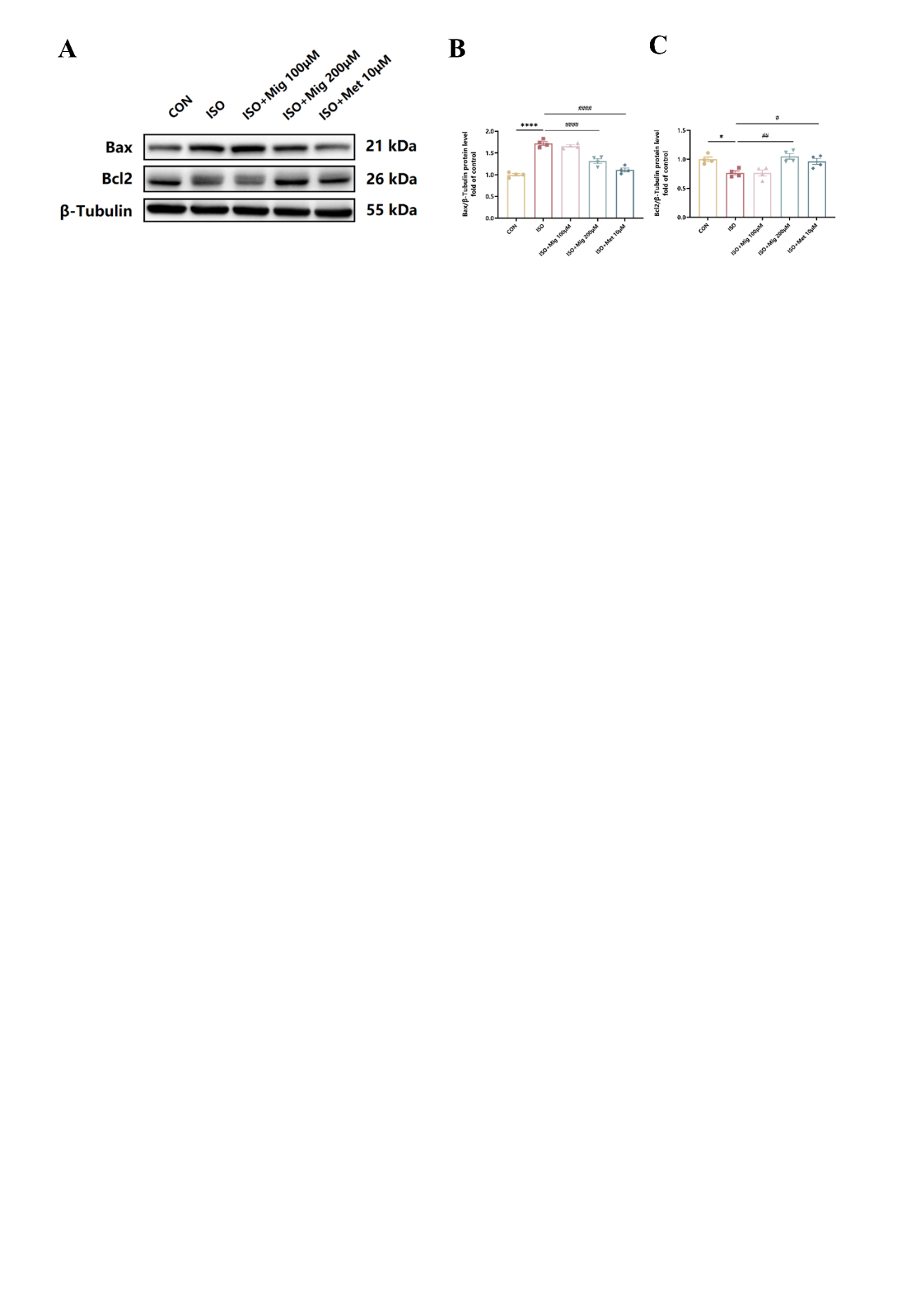


**Figure S5.** **Mig suppresses ISO-induced cardiomyocytes apoptosis in vitro.** (A) Representative images of Western Blot gel for Bax, Bcl2 signal pathways NRCMs. (B) Quantification of Bax on protein level in NRCMs (n=4 per group). (C) Quantification of Bcl2 on protein level in NRCMs (n=4 per group). Quantification of Bax and Bcl2 protein levels were normalized to β-Tubulin. The data were shown as Mean ± SEM (one-way ANOVA with Tukey’s post-hoc multiple comparison tests). *, P<0.05, ****, P＜0.0001 vs. CON; #, P<0.05, ##, P＜0.01, ####, P＜0.0001 vs. ISO.


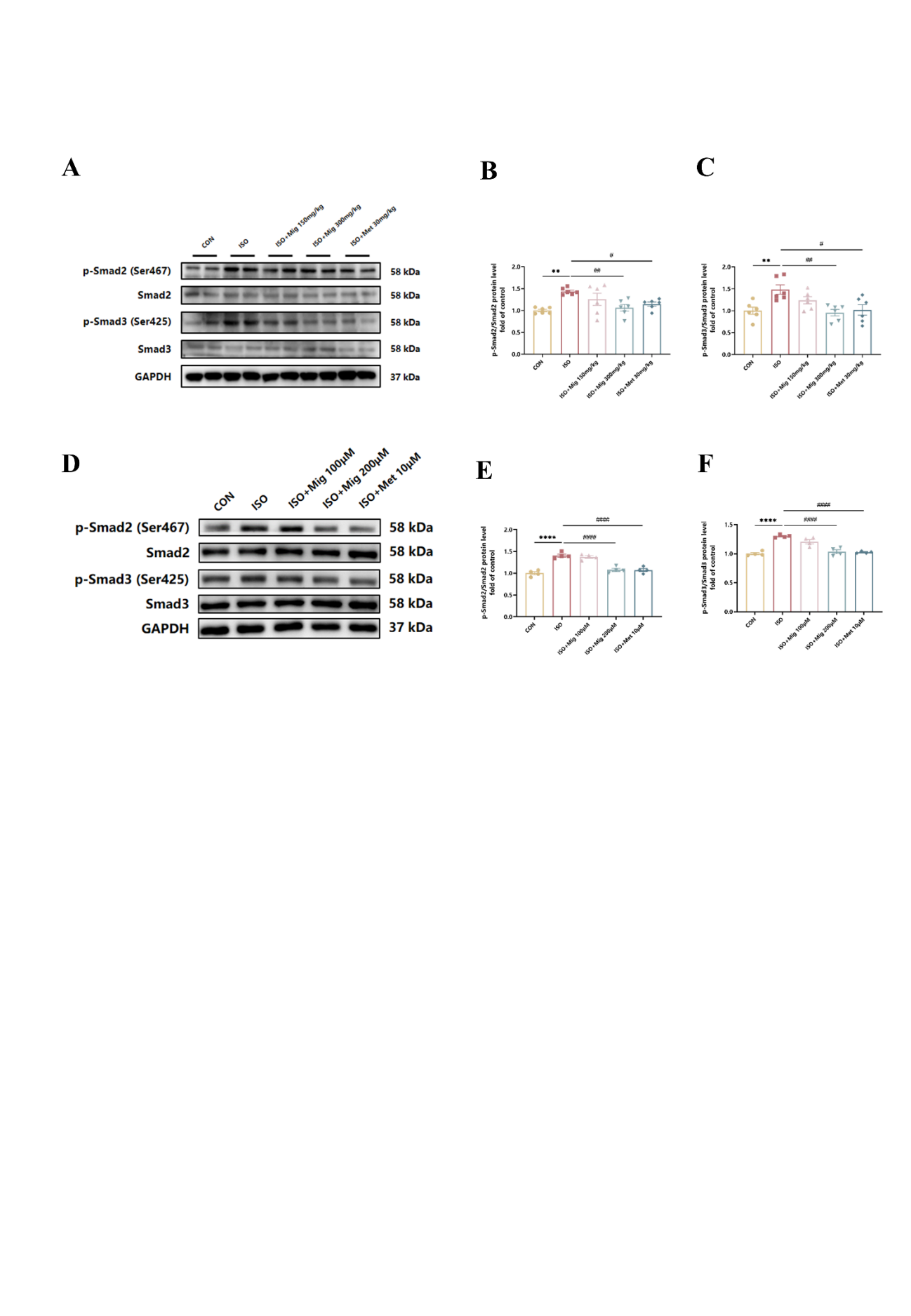


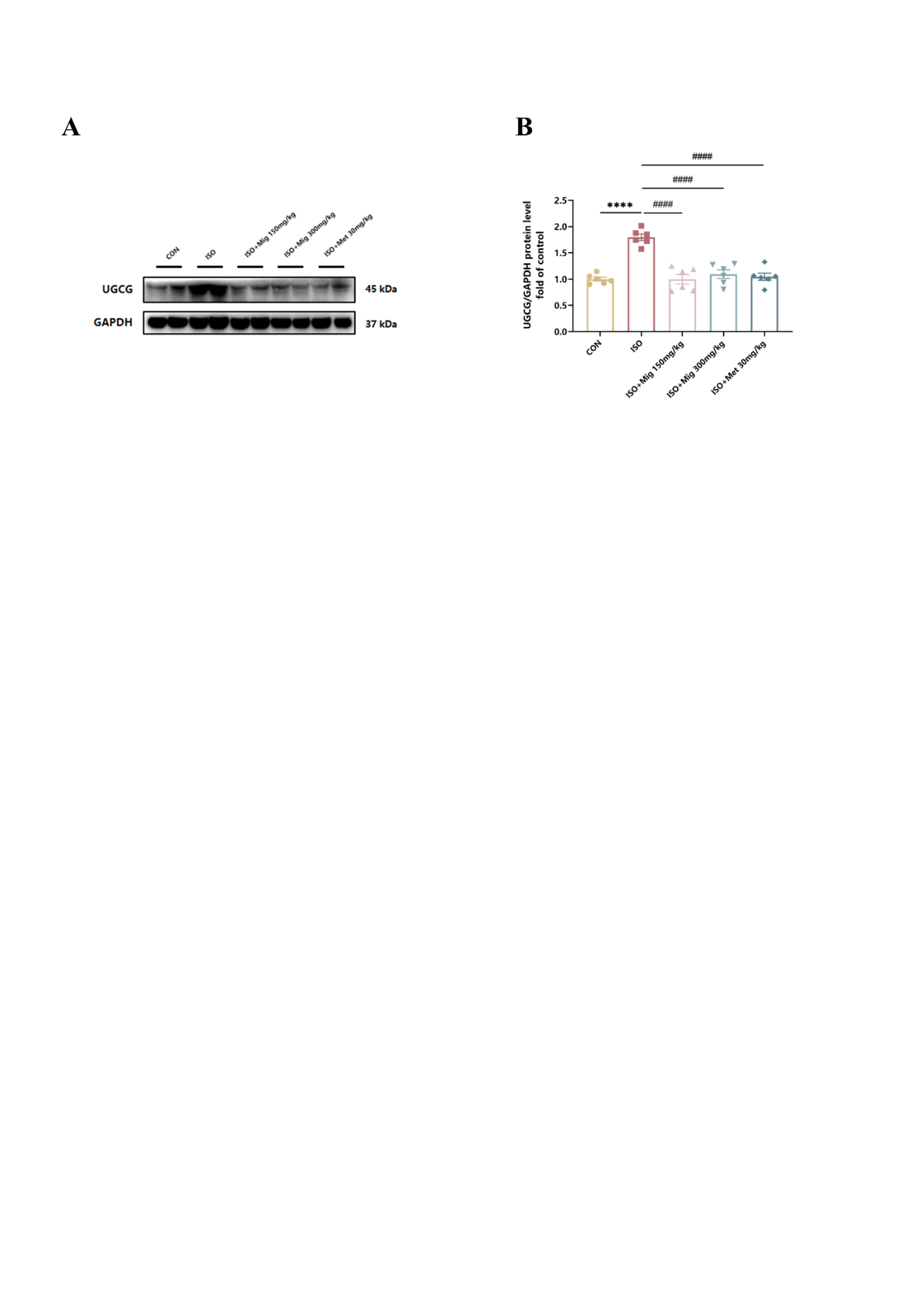
**Figure S6.** **Mig inhibits ISO-induced Smad2/3 signaling pathways.** (A) Representative images of Western Blot for p-Smad2/Smad2, p-Smad3/Smad3 in heart tissues. (B) Quantification of p-Smad2/Smad2 signaling in heart tissues (n=6 per group). (C) Quantification of p-Smad3/Smad3 signaling in heart tissues (n=6 per group). (D) Representative images of Western Blot for p-Smad2/Smad2, p-Smad3/Smad3 in NRCFs. (E) Quantification of p-Smad2/Smad2 signaling in NRCFs (n=4 per group). (F) Quantification of p-Smad3/Smad3 signaling in NRCFs (n=4 per group). The data were shown as Mean ± SEM (one-way ANOVA with Tukey’s post-hoc multiple comparison tests). **, P<0.01, ****, P<0.0001 vs. CON; #, P<0.05, ##, P＜0.01, ####, P＜0.0001 vs. ISO.

**Figure S7.** **Mig suppresses UGCG expression in ISO-induced cardiac fibrosis.** (A) Representative images of Western Blot for UGCG protein in heart tissues. (B) Quantification of UGCG protein level in heart tissues (n=6 per group). Quantification of UGCG protein level was normalized to GAPDH. The data were shown as Mean ± SEM (one-way ANOVA with Tukey’s post-hoc multiple comparison tests). ****, P<0.0001 vs. CON; ####, P＜0.0001 vs. ISO.
